# Pattern generation for direct-write three-dimensional nanoscale structures via focused electron beam induced deposition

**DOI:** 10.3762/bjnano.9.240

**Published:** 2018-09-27

**Authors:** Lukas Keller, Michael Huth

**Affiliations:** 1 Institute of Physics, Goethe University, Max-von-Laue-Str. 1, 60438 Frankfurt am Main, Germany

**Keywords:** focused electron beam induced deposition, nanofabrication, three-dimensional nanostructures

## Abstract

Fabrication of three-dimensional (3D) nanoarchitectures by focused electron beam induced deposition (FEBID) has matured to a level that highly complex and functional deposits are becoming available for nanomagnetics and plasmonics. However, the generation of suitable pattern files that control the electron beam’s movement, and thereby reliably map the desired target 3D structure from a purely geometrical description to a shape-conforming 3D deposit, is nontrivial. To address this issue we developed several writing strategies and associated algorithms implemented in C++. Our pattern file generator handles different proximity effects and corrects for height-dependent precursor coverage. Several examples of successful 3D nanoarchitectures using different precursors are presented that validate the effectiveness of the implementation.

## 1 Introduction

New physical effects and functionalities can arise when the third dimension can be accessed at the nanoscale. Geometrical and topological constraints present in lower dimensional structures can be overcome, as, e.g., coil-like or, more generally, chiral structures can be fabricated in 3D with relevance for metamaterials, such as in plasmonics [1–2]. Moreover, novel physics may arise, as is the case in nanomagnetic 3D structures which can, for example, show novel types of magnetic domain walls [3], or concerning magnetically frustrated interactions in 3D artificial spinice systems [4]. Being able to fabricate 3D nanostructures is thus beneficial for both the development of new technological applications and addressing more fundamental research questions.

Several sophisticated techniques have been developed to prepare 3D nanostructures, but fabrication without constraints on their shape and material composition remains an enormous challenge. One state-of-the-art approach to fabricated 3D systems on the nanoscale uses a layer-by-layer based technique [4]. The design of the desired 3D structure is partitioned into horizontal slices parallel to the substrate surface. For each slice a full set of structure-definition steps, typically combining physical vapor deposition and UV or electron beam lithography, are applied. This process is not only challenging due to the need for accurate alignment of all consecutive slices, but also extremely time consuming, depending on the required resolution in the vertical dimension. Other methods are two-photon lithography [5], heterogeneous nucleation [6] or template-based plating [7], to name a few.

In this work, focused electron beam induced deposition [8] (FEBID) is used as a mask-less direct-writing technique that allows for the deposition of structures with a resolution of less than 10 nm in 2D [9–10]. The working principle of FEBID is as follows: A substrate, or any other kind of solid support, is placed inside a scanning electron microscope (SEM). A precursor gas is supplied in close proximity to the focus of the primary electron beam. This is typically done by employing a gas injection system (GIS). The precursor molecules adsorb and diffuse on the substrate surface. As the focused electron beam is directed to predefined positions, chemical bonds of precursor molecules at these positions break, mainly via the generated low-energy secondary electrons (SE) [11]. The nonvolatile precursor fragments remain as deposits. Depending on the precursor used, and also on the chosen process parameters, a wide range of different materials can be obtained. On the one hand, polycrystalline metals can be realized, mainly of ferromagnetic type like Fe, Co or Fe–Co alloys [12–13] or noble metals, such as Pt, Au and Ag [14–16]. On the other hand, granular metals [17] but also various oxides and carbides in either amorphous or polycrystalline form [18–19] are accessible. By employing additional postdeposition treatments the metal volume content of some otherwise granular metals can be increased to virtually 100% [20–22].

Despite the apparent simplicity of the FEBID process, a more detailed look reveals a rather high degree of complexity. During a deposition event at a predefined beam position, precursor molecules are consumed, so that the precursor coverage on all exposed surface areas is space- and time-dependent. Since the deposition rate depends on both the available SEs and the available precursor molecules, the deposition rate also becomes space- and time-dependent. After some characteristic time, depending, e.g., on the precursor flux and the precursor type, surface diffusion leads to a replenishment in all of those regions where the precursor coverage has been reduced. Within an effective model approach, this has been very successfully described by a suitably adapted reaction diffusion equation, as recently reviewed by Toth et al. in the two-dimensional case [23]. In the 3D case, locally transient behavior in the precursor coverage becomes particularly critical since precursor transport via diffusion on the two-dimensional substrate surface is much faster than along the quasi-one-dimensional 3D edges of the growing 3D structure. Quite generally, the higher a deposit becomes, the slower the precursor transport via diffusion from the substrate to the current writing area. Additionally, deposition events in a given spatial region of the growing 3D deposit lead to a decrease of the total available precursor molecules in this region, thus also influencing the deposition rate in nearby regions (proximity effect). These effects have to be taken into account in order to deposit well-defined 3D structures that are in satisfying agreement with the originally targeted geometry.

Fowlkes and collaborators have demonstrated the potential of a simulation-guided and computer-aided design (CAD)-based approach to 3D nanofabrication via FEBID [24], which has already been shown to be very useful in obtaining plasmonically active, all-metal 3D nanostructures when combined with a suitably adapted postgrowth purification treatment [22]. Adopting general guidelines for optimizing the 3D writing strategy [22,24], we showed in a collaborative work that high-quality complex ferromagnetic 3D nanoarchitectures for studying magnetically frustrated systems can be fabricated by FEBID [25–26]. From a practical point of view, one needs to have a suitable pattern-definition file fed to the pattern generator of the SEM that controls the electron beam’s deflection and eventually leads to the growth of the desired 3D nanostructure. Ideally, this pattern-definition file should be generated quasi-automatically from a simple geometrical description of the target 3D structure.

A simulation-guided generation of pattern-definition files may well prove to be superior for some very demanding nanoarchitectures. We refer to [24] for details about 3D FEBID simulation. Nevertheless, at the same time, for complex and large target structures, an exact simulation is a computationally intensive task, whereas employing some computationally cheaper algorithms can yield pattern-definition files in a few seconds up to a few minutes on a standard PC. Especially for large structures, the 3D FEBID process becomes in many cases less challenging due to longer precursor replenishment times.

Fowlkes et al. [27] and Winkler et al. [28] have recently published CAD software to generate pattern files for 3D FEBID depositions. Here, we present our approach to generating such a pattern-definition file using some general rules of precursor dynamics. The algorithms, reflecting successful writing strategies as discussed below, are implemented in C++ for speed, flexibility and independence from other software with no dependence on nonstandard C++ libraries. In several important aspects, our approach differs significantly from [27–28] and we demonstrate that our code generates pattern-definition files that result in 3D deposits in very good correspondence to the targeted geometries. We therefore hope that our contribution may prove to be useful for a growing community of FEBID users targeting 3D nanoarchitectures for various application fields.

## 2 Algorithms

**Figure 1:**
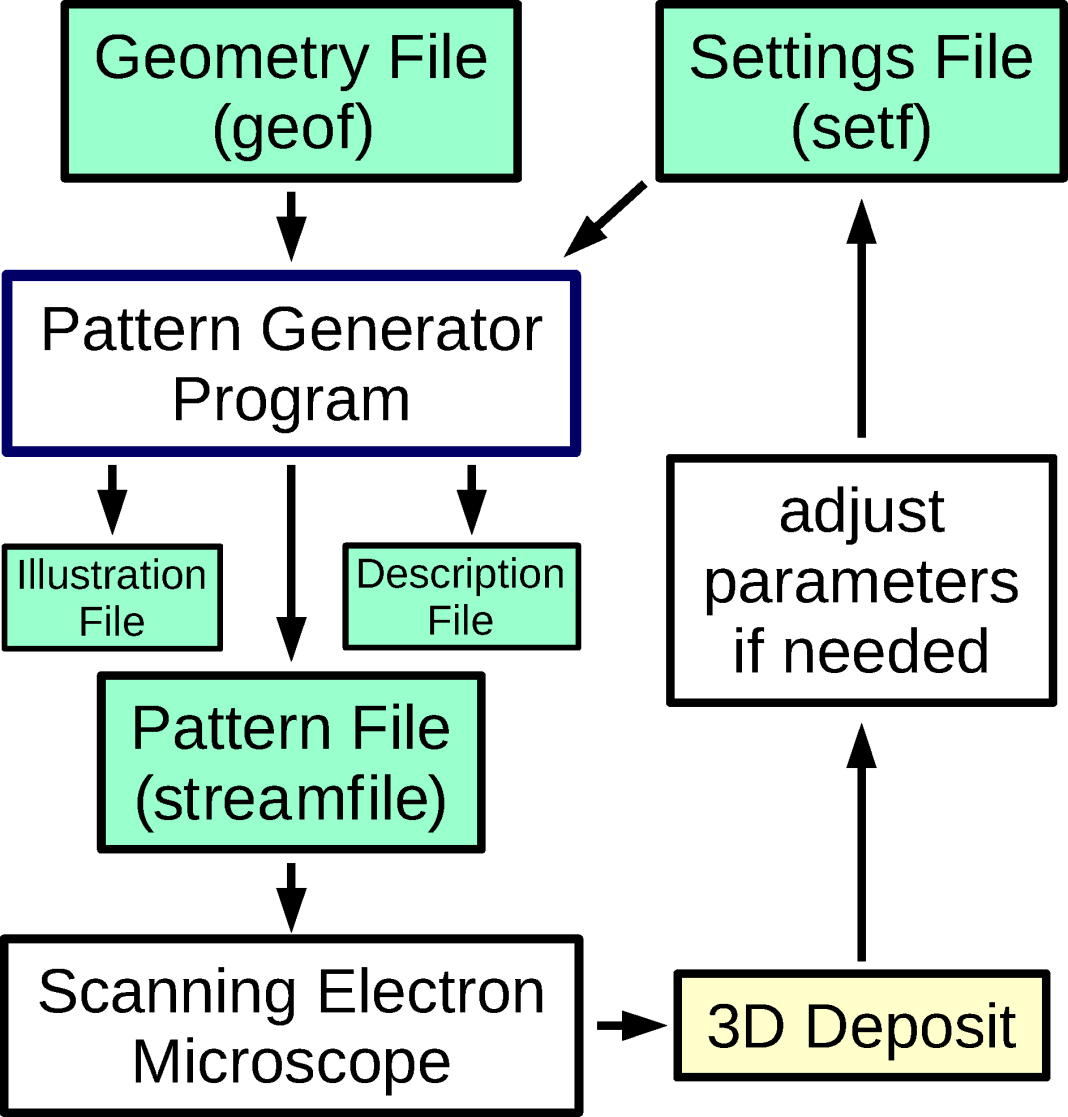
Block-flow diagram of the environment of the pattern generator program using input from a geometry description (geof) of the target 3D structure and process-specific parameters (setf). Besides the pattern file, a file for illustration purposes is generated which can be loaded by the program Gnuplot [29] in order to visualize the generated pattern. In addition, a description file, containing all used parameters for the pattern generation, is provided.

The algorithms require the geometry definition of the target 3D structure as input, which we consider as a wireframe. The geometry is provided in a file denoted as geof which contains the coordinates of a set of points in 3D space (vertices), as well as information on the lines connecting these points (edges). As code output one obtains a pattern-definition file (herewith denoted as pattern file; for FEI systems they are called streamfiles). The pattern file is loaded into the SEM’s pattern generator causing the electron beam movement that eventually leads to the desired 3D structure. The pattern file consists of a header followed by a table of the individual beam positions {*P*_2D_} = {*x*_2D_, *y*_2D_} and the dwell times {*t*_d_} for each of the positions. A block-flow diagram of the process is shown in Figure 1.

In the geometry file geof, a subset of the vertices is labeled as inital vertices, which represent the locations from which the algorithm should start to generate the writing sequence mapped to the pattern file. These initial vertices will conventionally be located at the bottom of the 3D structure, i.e., at *z* = 0 with the *z*-axis pointing opposite to the electron beam direction. In order to keep the code independent from any precursor- or process-specific parameters, a settings file (denoted as setf) needs to be supplied which contains all process- and precursor-specific parameters which depend also on the beam parameters (energy and current), and information about the geometry of the gas injection system (e.g., azimuthal and polar angles). All input and output files of the pattern generator program are explained in detail in the Supporting Information File 1.

As the code progressively goes through the generation steps for the pattern file, it needs to keep track of the state of the already processed elements of the geometry file. In order to do this, the code implements two main object types (classes): vertices and edges. A vertex has a 3D point location *P*_3D_ = (*x*_3D_, *y*_3D_, *z_3D_*) (in units of nm), a state variable which can hold the values ”reached” or ”not reached” and a list of all edges that terminate in the vertex. The state variable is set to ”reached” if all edges terminating in the point have reached the point. Every edge stores a reference to its start- and end-vertex, *P*_s_ and *P*_e_, keeps two state variables which can take on the values ”activated” or ”not activated” and ”finished” or ”not finished”, respectively, and keeps a variable *i*_e_ which stores the writing progress for the edge (see section 2.1). Each edge state variable is set to ”activated” when the edge’s start-vertex is reached and ”finished” when the respective end-vertex is reached.

Typically, the pattern generator of a SEM that controls the deflection of the electron beam uses the 2D coordinates *P*_2D_ referring to the horizontal field of view width (HFW) in some length unit (typically nm). Conventionally, a pattern generator accepts only positive integers ranging from 0 to 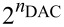 for every dimension, where *n*_DAC_ = 16 for our instrument (FEI Nova NanoLab 600). However, all calculations within the code are done with floating point numbers within the 3D space coordinate system. In this system a deposition event (DE) is represented by its location and duration, (*P*_3D_, *t*_d_). An entry into the pattern file relating to a specific deposition event (DE) is associated with a coordinate mapping in which the *z*-coordinate in 3D space has no relevance

[1]
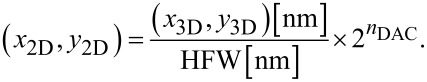


### 2.1 Overview of program flow

At initialization time, all vertex and edge information are loaded from the geometry file geof. All edges are automatically divided into a number of shorter edges (sub-edges), thereby introducing additional vertices. The length of a sub-edge has an upper limit *l*_max_ which is specified in the settings file setf. A value of 20 nm is typical. The edges’ state variables are set to ”not activated” and ”not finished”. The vertices’ state variables are set to ”not reached” if they do not correspond to initial vertices, in which case they are set to ”reached”. The subdivision of the edges has the disadvantage that it introduces new vertices, but it also allows for changing, e.g., the distance between two neighboring DE locations (also denoted as pitch Π_3D_ = (Π*_x_*, Π*_y_*, Π*_z_*)) or the duration of a dwell event *t*_d_, as the pattern file generation progresses from one sub-edge to the next. Within one sub-edge, pitch and dwell time are fixed.

The generation of the pattern file proceeds in a frame-to-frame fashion, where one frame contains one DE for each edge in state ”activated” and ”not finished”. An important variable for each edge object is the deposition speed *s*_F_. This variable subsumes the consequences of the edge’s angle relative to the substrate (inclination) and different growth-specific effects to be discussed in more detail in sections 2.2, 2.3.1 and 4.1. Its value specifies the length increase along the edge in units of nm per frame, and is calculated once for each sub-edge at initialization time. Another edge-related variable keeps track of the number of deposition events *n*_e_ necessary to write the edge. *n*_e_ represents the upper limit of variable *i*_e_ that monitors the writing progress for the edge. The value for *n*_e_ follows from the length of the edge *l*_e_ divided by the value of *s*_F_. Equation 2 provides an overview of the simple relationships between the variables

[2]
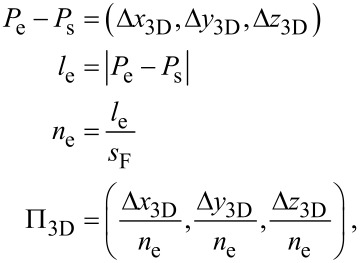


where Π*_z_* is just provided for completeness and has no further relevance.

**Figure 2:**
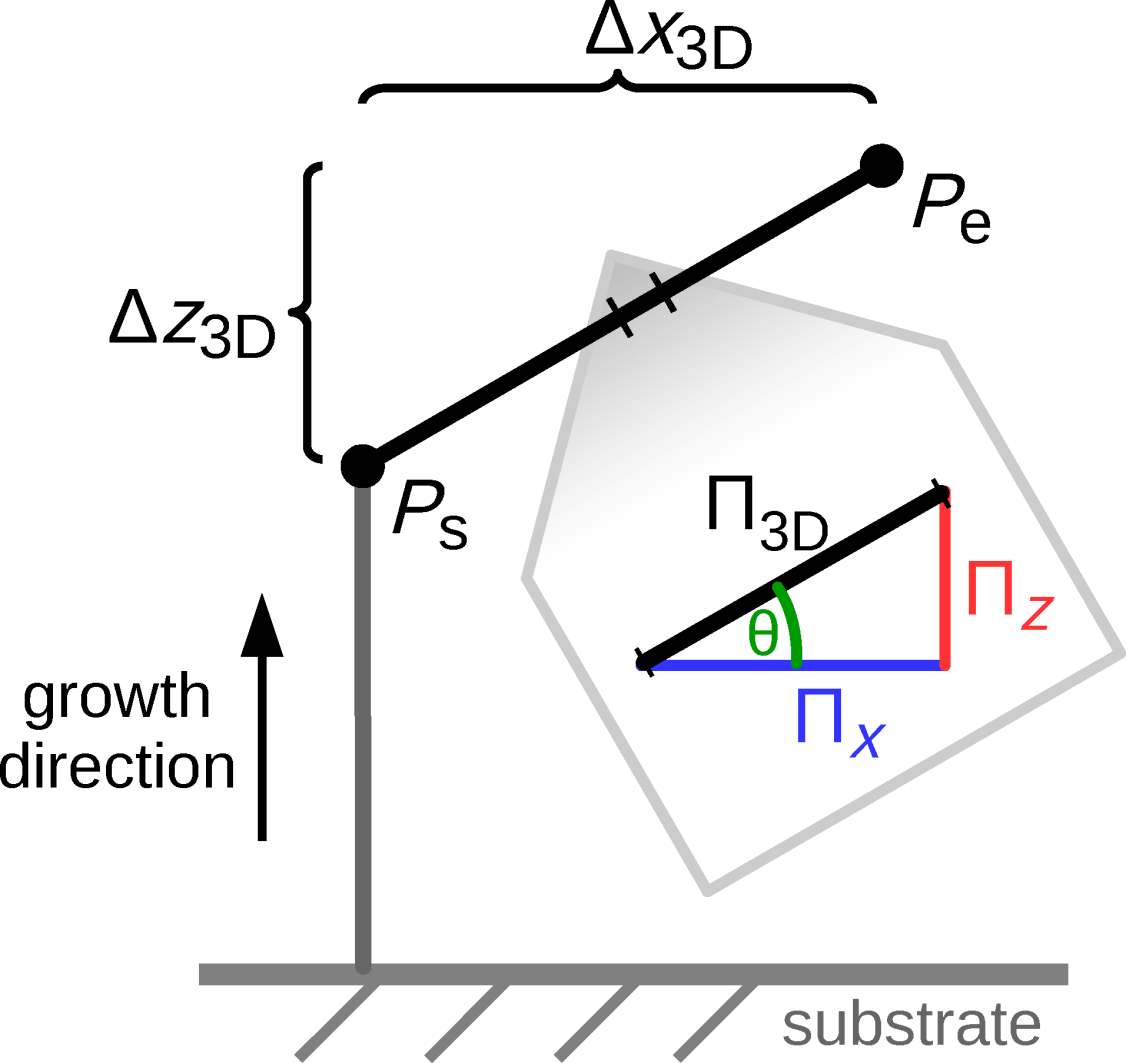
Illustration of the three dimensional pitch Π_3D_, projected in the *x*–*z*-plane: In every frame each active edge grows in the direction and with the length of Π_3D_. The electron beam moves every frame with Π_2D_, which is the projection of Π_3D_ into the *x*–*y*-plane. The direction of Π_3D_ is defined by the start point *P*_s_ and end point *P*_e_. Its length |Π_3D_| = *s*_F_ is determined by several parameters, like *x*_F_, *z*_F_ and the edge’s height.

After initialization the main loop of the program is entered. Each pass of the main loop generates one new frame containing one DE for each currently active edge. The position of a DE is calculated assuming a straight line between *P*_s_ and *P*_e_ of each edge and has a distance to the edge’s previous DE according to the initially calculated pitch Π_3D_, see Figure 2.

In a simple and mostly sufficient case, the duration *t*_d_ of a deposition event is set to a constant value, typically 1 ms. We note here that the deposition events within one frame have to be sorted in a suitable way in order to reduce proximity effects (see section 2.3.2).

As the deposition events (DEs) are processed, the corresponding state variables of edges and vertices are updated. The main loop is repeated until every vertex reads ”reached” in its state variable. At the end of the program the pattern file is written to the hard drive, as well as a simple control file to be loaded by the command-line-driven graphics utility program Gnuplot [29] that illustrates the generated pattern file. For reference, an additional description file is created that lists all parameters used.

### 2.2 Deposition speed *s*_F_ and edge inclination dependency

As stated before, in every frame the current length of each active edge increases by its *s*_F_ in the direction of its Π_3D_ (see Figure 2). For a relatively short pillar, with inclination angle θ = 90° towards the substrate, *s*_F_ becomes the vertical growth rate *z*_F_ for the given experimental parameters (precursor type, beam parameters, gas flux and direction, substrate material, etc.). *z*_F_ can be easily calibrated by writing a pillar with the height *h*_def_ specified in the geof, such as *h*_def_ = 300 nm, and an educated guess for the value of 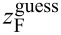 (e.g., 0.05 nm per frame). After pillar growth, the factual height *h*_meas_ of the deposit has to be measured (e.g., 200 nm). The calibrated value for *z*_F_ is then

[3]
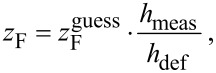


in our example *z*_F_ = 0.033 nm per frame.

As a first guess one might assume *s*_F_ ≈ *z*_F_ to be a reasonable choice, independent of the edge inclination angle θ. However, the actual edge angle formed depends on the time-averaged ratio of secondary electrons leaving the already deposited edge’s top and side surface. Apart from some material and beam properties, which are fixed during deposition, this ratio is determined by the exact shape of the already deposited edge and the applied deposition speed *s*_F_. Moreover, the deposit volume which is penetrated by the electron beam becomes smaller the flatter an edge is, which influences the amount of available secondary electrons. Because of this, in general *s*_F_ ≠ *z*_F_ for nonvertical edges.

Winkler et al. and Fowlkes et al. [27–28] realized the necessity to adopt the velocity of the (two-dimensional) beam movement with respect to the edge inclination. Their approach is to deposit a number of edges with increasing beam displacement velocity and to take the measured edge inclination angles as calibration data. Based on this calibration data, for each deposit edge in the target structure, an appropriate beam displacement velocity is chosen.

We use a different approach based on the three-dimensional pitch Π_3D_, as stated before (see Equation 2). As the 3D deposition speed *s*_F_ has to be adapted for nonvertical edges, in particular for horizontal edges, we introduce the additional parameter *x*_F_ that represents the deposition speed for horizontal edges. We do not assume any dependence of the growth speed on the azimuthal angle, so there is no need for a *y*_F_. Consequently, the pattern generator takes as the deposition speed of an edge in the two limiting cases

[4]
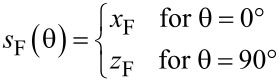


For interpolating between these two values, ideally the exact geometry of the tip of an edge (see Figure 15, to appear later) should be taken into account to calculate the ratio between the amount of secondary electrons leaving the edge’s surface at the top and at the side. However, we have been successful by implementing a linear interpolation (Manhattan distance) according to


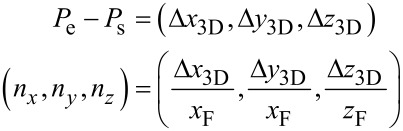


and

[5]
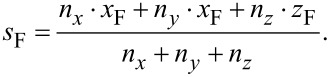


A quadratic interpolation (Euclidean distance)

[6]
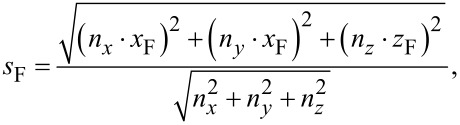


which was our first guess, did not perform as well.

Both Equation 5 and Equation 6 respect Equation 4 with *n*_x_ · *x*_F_ = Δ*x*_3D_ = *l*_e_ · cos(θ) and *n*_z_ · *z*_F_ = Δ*z*_3D_ = *l*_e_ · sin(θ). A deposition series with various edge angles is shown later in Figure 7. A deposition for calibrating *x*_F_ and *z*_F_ is also shown later in Figure 6.

### 2.3 Proximity corrections and handling of nonstationary growth conditions

The local deposition rate in a DE area depends on the precursor density and the secondary electron flux density for a given effective dissociation cross section [8,30]. The most simple implementation of code for pattern file generation would be to process the vertex-edge information in the geometry file in a linear sequence. However, the resulting deposit geometry will satisfy the target structure only when, for a given DE the precursor coverage is stationary at the writing position, i.e., when all DEs occur within the electron-limited growth regime of FEBID [22]. This is, however, often not the case – in particular for simple structures with short loop times – and the precursor coverage is time- and space-dependent, and is also particularly influenced by previous DEs that took place in close proximity (see section 2.3.2). Local precursor replenishment following a DE occurs by direct local adsorption and by diffusive transport from areas with larger precursor coverage [22]. Although direct local adsorption can be hindered by shadowing of directed precursor flux from the GIS caused by an already existing 3D deposit, this effect was found to be of minor relevance in the experiments carried out in this work. Nevertheless two algorithms for avoiding shadowing of direct precursor flux have been implemented (see section 4.2 and 4.3), which can be activated if required.

Diffusive precursor transport occurs along the existing 3D structure tapping into the large reservoir formed by the precursor coverage of the large-area substrate. Conceptually, the precursor conductance by diffusive transport along an edge scales linearly with the edge diameter and the inverse of the edge length. The conductance of several edges in parallel sum up, whereas it is the sum of the inverse of the conductances that add if several edges are arranged in series. Accordingly, with increasing distance from the substrate surface, the supply of growing edges with precursor tends to get smaller, but in a way that depends on the topology and geometry of the growing 3D structure. In our algorithmic implementation this is taken into account by the edge-specific variable *s*_F_ (see section 2.3.1).

#### 2.3.1 Height-dependent precursor supply

As the growth proceeds, the *z*_3D_ values of the deposit increase and the local precursor coverage decreases due to the decreasing diffusive up-flow from the substrate surface. Since the local growth rate is proportional to the precursor coverage, measures have to be taken to compensate for this effect. The most accurate and general solution is to repeatedly simulate the precursor coverage for small time intervals on the substrate surface and 3D deposit [24] and use the gained information to adapt the deposition speed *s*_F_. This is a computationally intensive task. Here we follow a different approach based on an initial deposition experiment for calibration.

**Figure 3:**
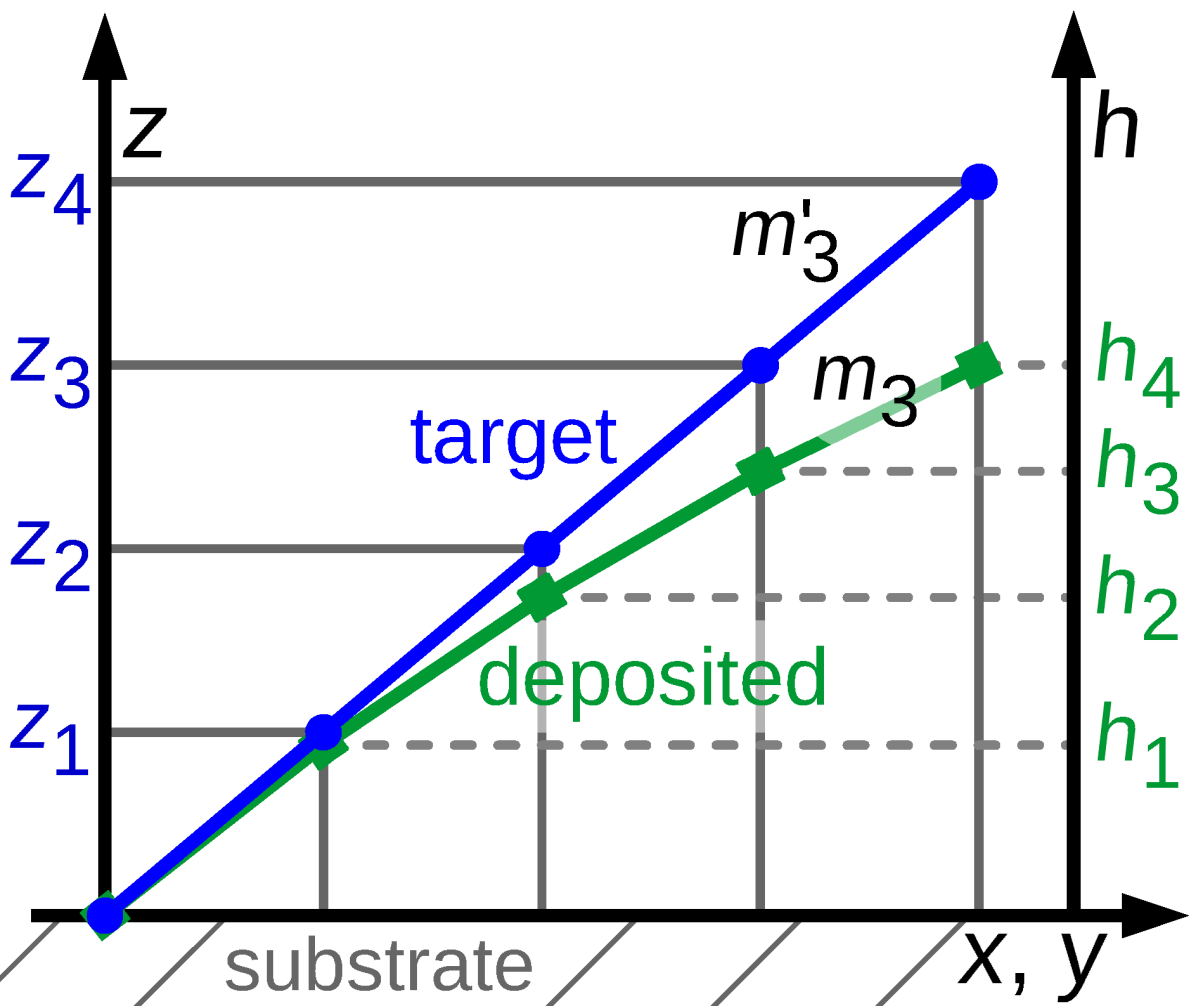
Visualization of the height-dependent deposition rate: The blue circles correspond to vertices defined in the geometry file with the desired height *z*_3D,_*_i_*. The green squares represent data taken from a deposited structure via its SEM picture with the measured height *h_i_*. 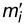 stands for the desired slope of a given edge, *m_i_* for the slope at a given position of the actual deposit. The higher a deposited edge becomes, the lower gets the actual deposition rate (with fixed parameters) due to a lower precursor replenishment by diffusion from the substrate.

First, the target 3D structure is deposited by using the pattern file generated by the code with the height-dependent deposition speed correction deactivated (see below). Next, from SEM inspection of the deposit the heights {*h_i_*} of a small set of locations sufficiently remote from the substrate surface are measured. When compared to the target heights {*z*_3D,_*_i_*} of the respective locations one will in many cases find that the slope *m_i_* between two next-neighbor heights of the deposit *m_i_* = (*h_i_*_+1_ − *h_i_*)/(*s_i_*_+1_ − *s_i_*) is smaller than the expected slope 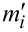 from the target heights 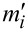 = (*z*_3D,_*_i_*_+1_ − *z*_3D,_*_i_*)/(*s_i_*_+1_ − *s_i_*). Here we have introduced the lateral coordinates denoted as *s_i_*. They are not important as will become clear shortly. The observation *m_i_* *<* 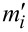 is a direct consequence of the reduced precursor coverage at larger *z*_3D_ values. Within our code this is now taken into account by adjusting all of the edge variables *s*_F_ by a height-dependent correction factor η(*z*_3D_). This is done at initialization time and before the code enters its main loop. How this correction function is obtained from the slopes {*m_i_*} and {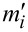} is described next. For visualization of the height-dependent deposition rate see Figure 3.

Renormalizing the variable *s*_F_ for each edge with average height ζ = (*P*_e_.*z*_3D_ + *P*_s_.*z*_3D_)/2 by the factor *m*/*m*^′^ determined from the slope ratios *m_i_*/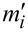 found in the calibration experiment at a similar height *z*_3D,_*_i_* ≈ ζ would compensate for the reduced precursor coverage effect because *n*_e_ = *l*_e_/*s*_F_. Now we have


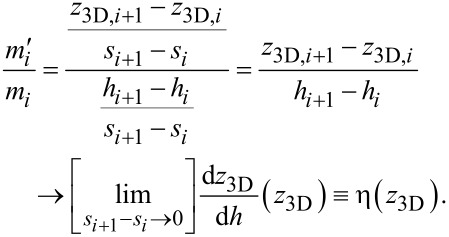


For the set of values {(*h_i_*_+1_ − *h_i_*)/(*z*_3D,_*_i_*_+1_ − *z*_3D,_*_i_*)} vs the heights {*z*_3D,_*_i_*} obtained from the calibration experiment we perform a polynomial fit and obtain


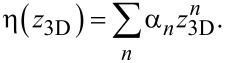


*n* ≤ 3 is sufficient, since for the most simple 3D structure – a vertical pillar – the stationary state solution of the diffusion equation leads to a linear decrease of the precursor coverage with growing *z*_3D_ value. For more complex structures this dependence will in general turn out to be nonlinear but will certainly be described quite well by a low-order polynomial. The values of the coefficients α*_n_* are stored in the settings file setf. This implies that setting the coefficients to α_0_ = 1 and α*_n_* = 0 for all *n >* 0 does deactivate the height-dependent deposition speed correction. At initialization the *s*_F_ renormalization is now done according to


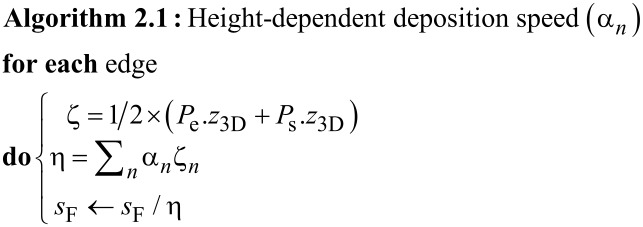


If the structure is reasonably regular and symmetric this approach works well. For structures with strong height-dependent variations in topology or geometry one set of parameters α*_n_* independent of the height will not suffice. In section 4.4.2 we discuss how to go beyond the present approach. The working height correction is demonstrated in Figure 10, to appear later.

#### 2.3.2 Proximity effects

A DE consumes precursor molecules. Any close-by DE will be affected by this precursor consumption if it is located within the area of the previous DE or is subject to diffusive drain of precursor because of the precursor depletion caused by the previous DE. There are two possible strategies to deal with this problem: compensate for it or avoid it as far as possible.

Within our approach a compensation algorithm implies a suitable renormalization of *s*_F_ for all DEs in a time-dependent fashion. For a fully satisfying solution this naturally leads again to a simulation-assisted algorithm. Here we follow a simpler strategy which works on a frame-to-frame basis and needs no preprocessing of the geometry file. Conceptually one would like to sort the DEs in one frame such that the time period between any two DEs which are characterized by closely spaced locations is maximized. We have implemented two different algorithms in order to avoid proximity effects.

**Figure 4:**
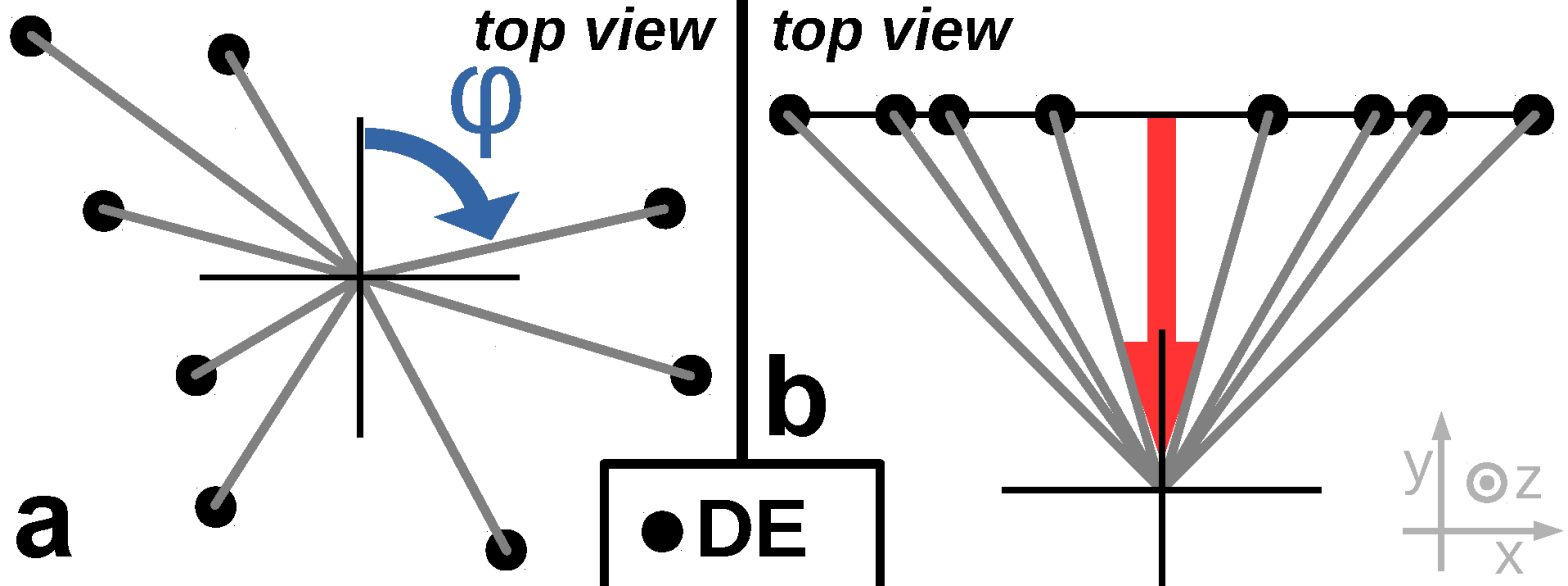
Schematic overview of the angle-sorted proximity avoiding algorithm (asPAA). a: In order to minimize the proximity effect the angle φ between a deposition event’s position (DE) and the center of all DEs of one frame is calculated for every DE. The final order of the DEs of a frame is chosen in order to let the electron beam move as far as possible between the DEs and not let it come back close to the DE for the next but one DE. b: If all DEs are in an area with a too high aspect ratio (e.g., bigger 1:10), like a line, the center of the frame’s writing area is artificially shifted in the shorter direction in order to have other φ than {φ_1_, φ_1_ + 180°}, which would produce a quite random order.

One approach is to generate many possible orders of DEs for each frame and analyze which performs best with regard to minimizing the proximity effect. This yields good results but is computationally expensive and not necessary for all geometries. First we describe a computationally cheaper solution:

From the locations {*P*_3D_} of all DEs to be processed in a given frame (F) the barycenter *P*_F_ is calculated


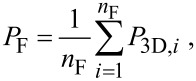


where *n*_F_ is the number of DEs in the frame. Next the DEs in F are sorted with regard to the azimuthal angle φ enclosed by the line connecting the locations of each of the DEs with the barycenter and the *y*_3D_ axis (see Figure 4). From the thus sorted set of DEs the first and then every (*n*_F_/*n*) modulo *n*_F_-th DE is processed (typically *n* ≥ 3) until the frame is completed. By this, e.g., for *n* = 3 a triangular-like pattern within a frame is achieved. Special care has to be applied if the locations of the DEs within one frame happen to be close to a linear arrangement or even fall on a line. In this case the azimuthal angle values separate into two groups about some values φ_1_ and φ_2_ ≈ φ_1_ + π. In this case *P*_F_ is shifted in the direction perpendicular to the linear arrangement (see Figure 4b) and the azimuthal angles are calculated again, followed by the sorting and DE processing as described above. Here we briefly show the pseudocode for handling the linear arrangement problem if the line orientation is either parallel to the *x*_3D_ or *y*_3D_ axis


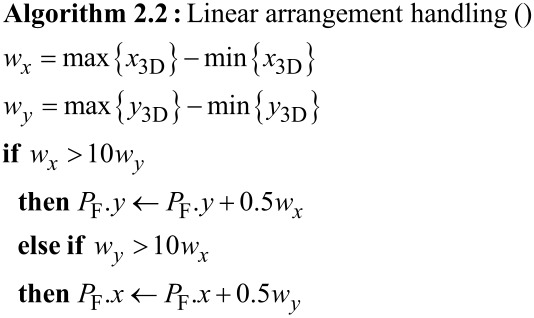


This angle-sorted proximity avoiding algorithm (asPAA) works well for highly symmetric structures for whose geometry the assumption is justified that a big difference in the calculated azimuthal angle φ of two DEs correspondents to a big distance between these DEs. An example of a successful deposition with the asPAA can be found in Figure 10, to appear later.

**Figure 5:**
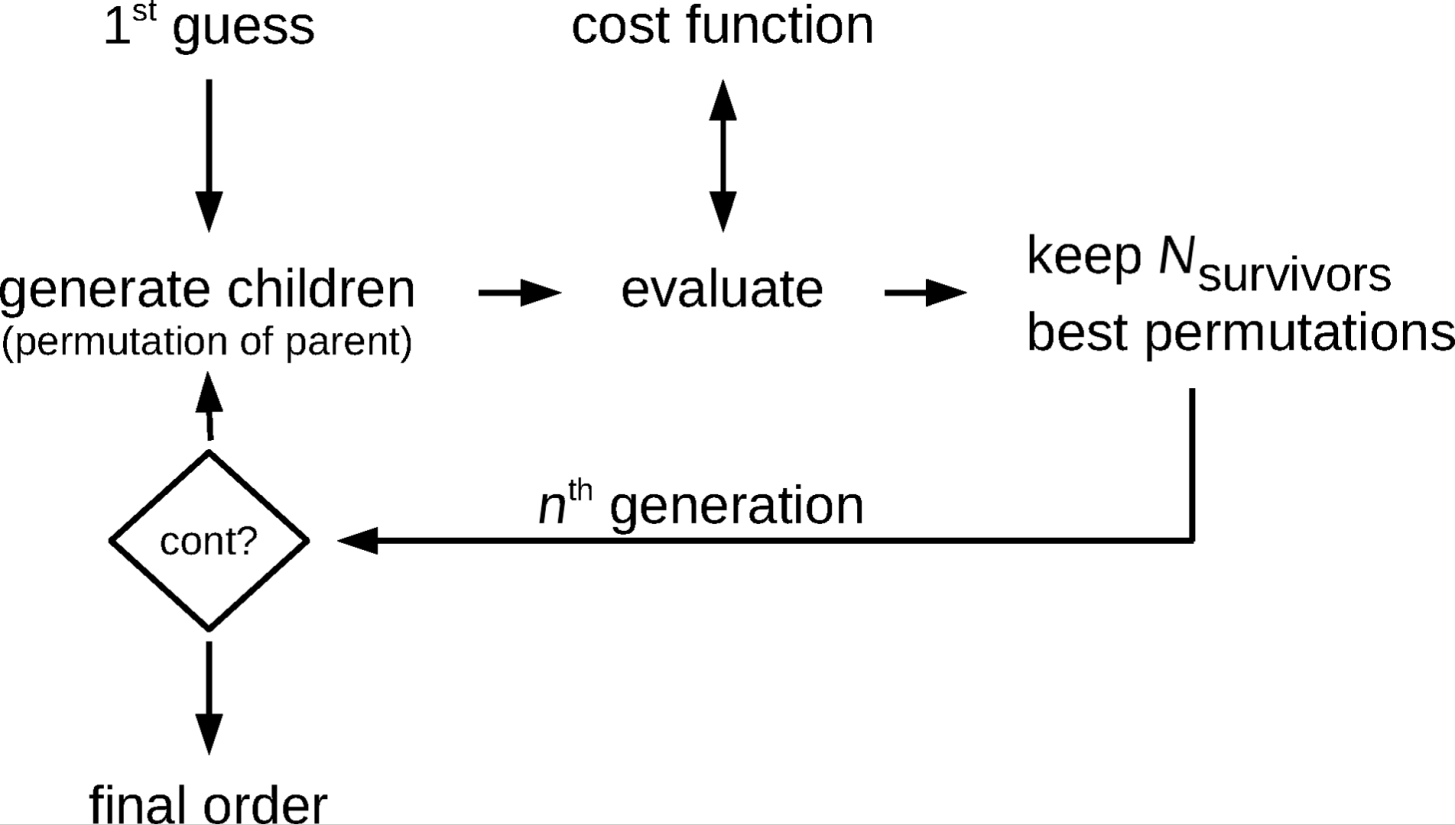
Schematic overview of the best permutation proximity avoiding algorithm (bpPAA). The algorithm generates new generations of possible orders of the DEs of one frame by building permutations of already known possible orders. Each order is evaluated by a cost function and the best order found will be employed for the current frame. As first guess the result of the angle-sorted PAA can be used. The actual cost function is chosen as the sum of Gaussians with the distance of two subsequent DEs as argument for each DE.

Figure 5 gives an overview of the more advanced proximity avoiding algorithm, which tries to find the best order of DEs within one frame. It takes a first guess of the order of the DEs in a frame. The program can be asked to perform the angle-sorted proximity algorithm (asPAA) first, in order to use its result as the first guess for the best permutation algorithm (bpPAA). The jump from one DE to the next is evaluated by a cost function. In general, the cost function could take many input parameters, such as the spatial distance between the two following DEs and their topological relation (e.g., are they connected by the same precursor supply chain to the substrate?) and calculates the ”badness” or ”cost” of any given transition between two following DEs. The algorithm detects the worst transition and generates all possible nontrivial permutations of the current order in which the positions of both bad DE’s are exchanged with all other positions of the frame. The total badness or cost of an order of DEs is the sum of costs of all DE transitions. All new orders are called the children of the parents (in this case of the first guess) and all parents and children form together the *n*-th generation of orders of this frame. The permutations are sorted by their badness and the best *n*_survivors_ orders form the parents for the next generation. The process finishes after a given number of generations. The best order of the final generation is taken as the final order for the DEs of the current frame.

The cost function used in this program only takes the spatial distance between two consecutive DEs as input. Small distances should result in a large cost. For larger distances the calculated cost should drop. For very large distances the cost should not decrease significantly since there is no improvement in avoiding the proximity effect by jumping very far distances. But a too positive evaluation for a very large jump at some time could then compensate too short jumps between two following DEs later on. In the current work a Gaussian is used with the distance of two consecutive DEs as argument. Its width can be defined in the settings file setf.

In general, also the choice of the dwell time *t*_d_ will influence the proximity effect. Long dwell times will result in an increase of the time between two successive DEs which can be beneficial in avoiding proximity effects. However, at the same time long dwell times reduce the time-averaged deposition rate per DE because of precursor depletion. In addition, the resolution may suffer due to charging issues caused by the electron beam. Conversely, short dwell times increase the time fraction of the total process time in which the electron beam is not placed at a writing position but moved in between two consecutive writing positions due to the limited deflection speed of the electron beam. This is an issue for SEMs with a magnetic scan system. In principle, the cost function could increase again for very large distances in order to prevent problems caused by too slow deflection speeds. However, we did not find any evidence in our deposits that we suffer from a too slow deflection speed. This might be also the case since quite long dwell times (1 ms) are used, compared to typical dwell times used for 2D FEBID depositions. We therefore did not implement a raising cost function for larger distances. A comparison of examples for the asPAA and bpPAA is shown in Figure 9, to appear later.

#### 2.3.3 Deposition of 3D heterostructures

A whole range of new applications becomes feasible if the patterning algorithm is extended to fabricate 3D heterostructures. This, however, introduces additional complexity in the deposition process, which we briefly discuss before presenting the implemented solution.

In general, each substructure made from one material has to be fabricated from its own pattern file and will require a different GIS with associated GIS settings. Also, before resuming deposition after changing the GIS setup a pumping period of sufficient duration has to be maintained in order to ensure that residual precursor from the previous deposition stage has been removed. Indeed, it may be necessary to introduce several pump and flush cycles with an inert gas. During the pumping (and flushing) time, and as a consequence of the GIS setup change, a shift of the writing location will likely occur that has to be compensated for, since already a mismatch of a few nm can compromise the complete 3D structure.

In our implementation, for the alignment between several fabrication steps (in particular between changes of the precursor) dedicated 2D auxiliary objects are defined in the geometry files. These will appear as first entries in the pattern files and will thus precede the beginning of the 3D deposition properly. The auxiliary objects comprise (a) four single dots written with a short dwell time at fixed positions defining the edges of a rectangle at the outermost corners of the field of view. By these markers, which have to be identical in all geometry files associated with the 3D heterostructure, the center of the writing field for each pattern can be matched using the SEM control software. This can be done manually or automatically, depending on the SEM software available. Additional auxiliary objects are (b) four- and eight-armed stars which, if defined in the geometry file, will appear before the 3D pattern definitions in the pattern file. These can be used for manual or automatic fine alignment by repeated image acquisition of a previously deposited pillar and corresponding beam shift before the actual 3D deposition commences. Special care has to be taken to minimize parasitic deposition during image acquisition for this fine alignment. An example of a successfully deposited two-material 3D structure (ferromagnetic Co_3_Fe and paramagnetic, nanogranular Pt(C)) involving three deposition steps is shown in Figure 13, to appear later.

## 3 Results

We now present the results of selected deposition experiments which have been performed for both determining the parameters in the settings file setf and testing the generated pattern files for various geometry files geof with different optimizations dis- or enabled, as discussed in section 2.3.

All experiments were performed with a dual beam microscope of type FEI Nova NanoLab 600 equipped with a Schottky emitter and operating at a base pressure of 3 × 10^−7^ mbar. Typical beam voltages and currents were 20 kV and 40 pA if not stated otherwise. Both the normal and high-resolution mode were employed. Two gas injection systems were used for the precursors, Me_3_CpMePt(IV) and HCo_3_Fe(CO)_12_ operating at 45 °C and 65 °C, respectively. The GIS positions were 100 μm above and 100 μm lateral offset to the centered beam position for both Me_3_CpMePt(IV) and HCo_3_Fe(CO)_12_. The polar/azimuthal angles were −60°/45° and −60°/−39°, respectively. All depositions were done on p-doped Si wafers with thermally grown SiO_2_ of 200 nm thickness. Au/Cr contacts, as used for some deposition experiments, were grown by sputtering to a thickness of 30 nm and 3 nm, respectively. The patterning was done by UV lithography using allresist AR-U 4040 and lift-off. In section 3.5 we state some of the execution times for the generation of the pattern files used for deposit fabrication as presented next.

### 3.1 Edge-angle-dependent deposition speed

**Figure 6:**
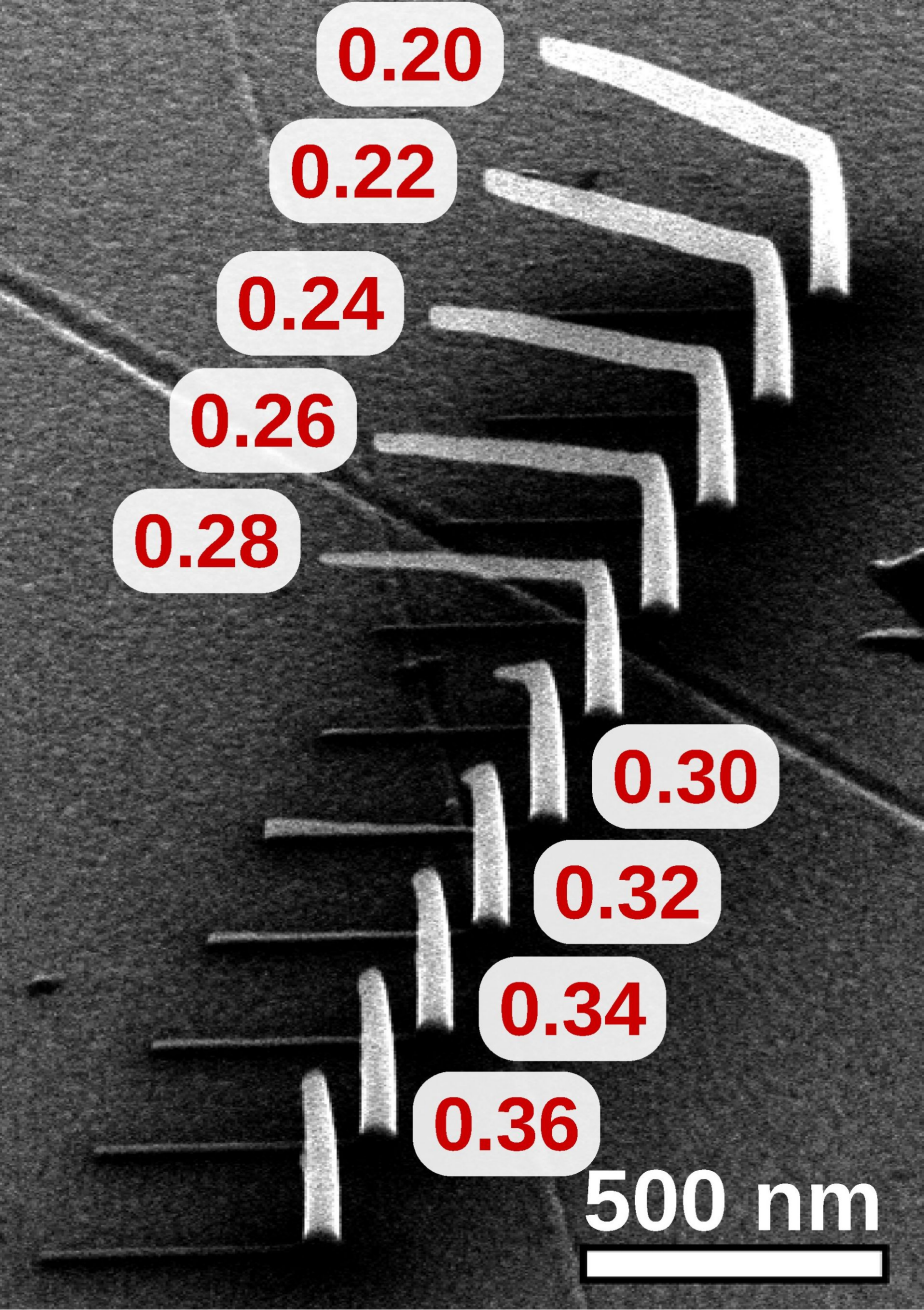
Calibration of deposition speed parameters *x*_F_ und *z*_F_. Shown is one deposition series from 52° tilted view, made up of several elements where each consists of a vertical pillar and an edge which is defined with a horizontal inclination in the geof. For every horizontal edge an individual *x*_F_ was used by a special command in the geof. The *x*_F_ values are displayed next to each element and range in this example from 0.20 to 0.36 nm per frame. This deposit was written in normal mode with 20 kV, approx. 40 pA using the precursor Me_3_CpMePt(IV). In this example the best value for *x*_F_ is between 0.28 and 0.30. In order to calibrate *z*_F_ the measured height of the vertical pillars has to be evaluated, see Equation 3. Here *z*_F_ was 0.135 nm per frame.

**Figure 7:**
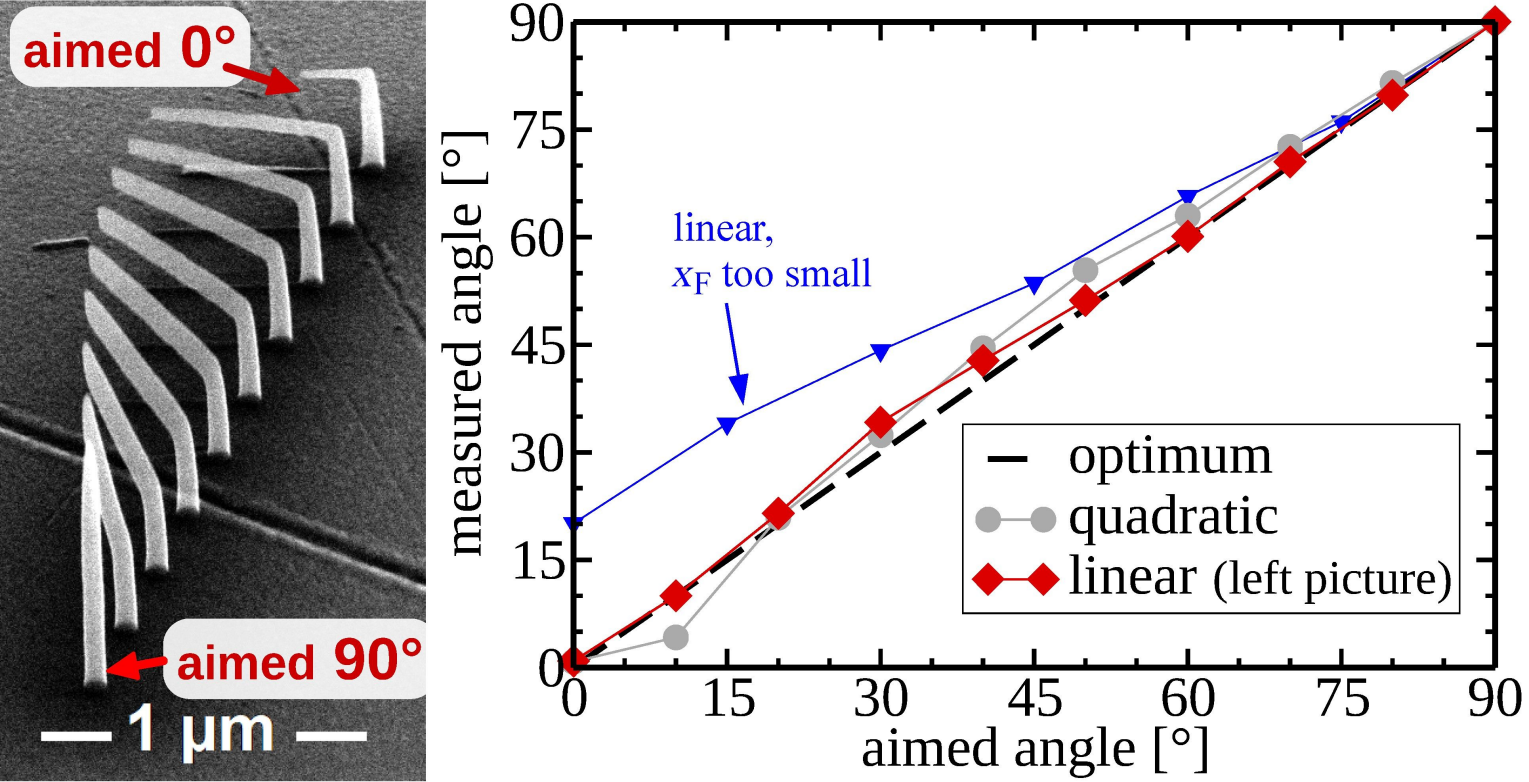
Angle-test structure consisting of several elements. Each element has a vertical pillar and a tilted edge with an inclination angle ranging from 0° to 90°. In the right plot the measured angles are plotted against the target angles for samples with the linear and quadratic interpolation function for *s*_F_. The best result is obtained with the linear interpolation. For this, the biggest mismatch is at the targeted angle of 30°, where instead 34.2° were measured. The measured angles are taken from 52° tilted SEM images. Precursor: Me_3_CpMePt(IV), normal mode.

In order to calibrate the deposition speed parameters *x*_F_ and *z*_F_, introduced in section 2.2, pitch-calibration structures, like the one shown in Figure 6, have been deposited. Each element consists of a vertical pillar and a horizontally defined edge at the top. Although all elements of the series were deposited simultaneously, for each horizontal edge another *x*_F_ was taken. This can be achieved by extra commands in the geof. After finding an appropriate value for *x*_F_ and *z*_F_, an angle-test structure, such as shown in Figure 7, can be deposited for verification. It consists of an array of vertical pillars with a tilted edge connected at the top. The angles of the tilted edges range from 0° to 90° and are defined in the geof. Note that the values for *x*_F_ and *z*_F_ were now fixed for the whole deposition series. Note also that there is no need to deposit the angle-test structure each time after suitable values for *x*_F_ and *z*_F_ have been found.

The result shown in Figure 7 has been achieved by using just the parameters *x*_F_, *z*_F_ and a very simple, inclination-angle-dependent interpolation function for the growth speed *s*_F_ (see Equation 5). As already stated by Winkler et al. [28] the deposition of flat edges is challenging and depends also on the quality of the electron beam focus.

### 3.2 Proximity corrections

**Figure 8:**
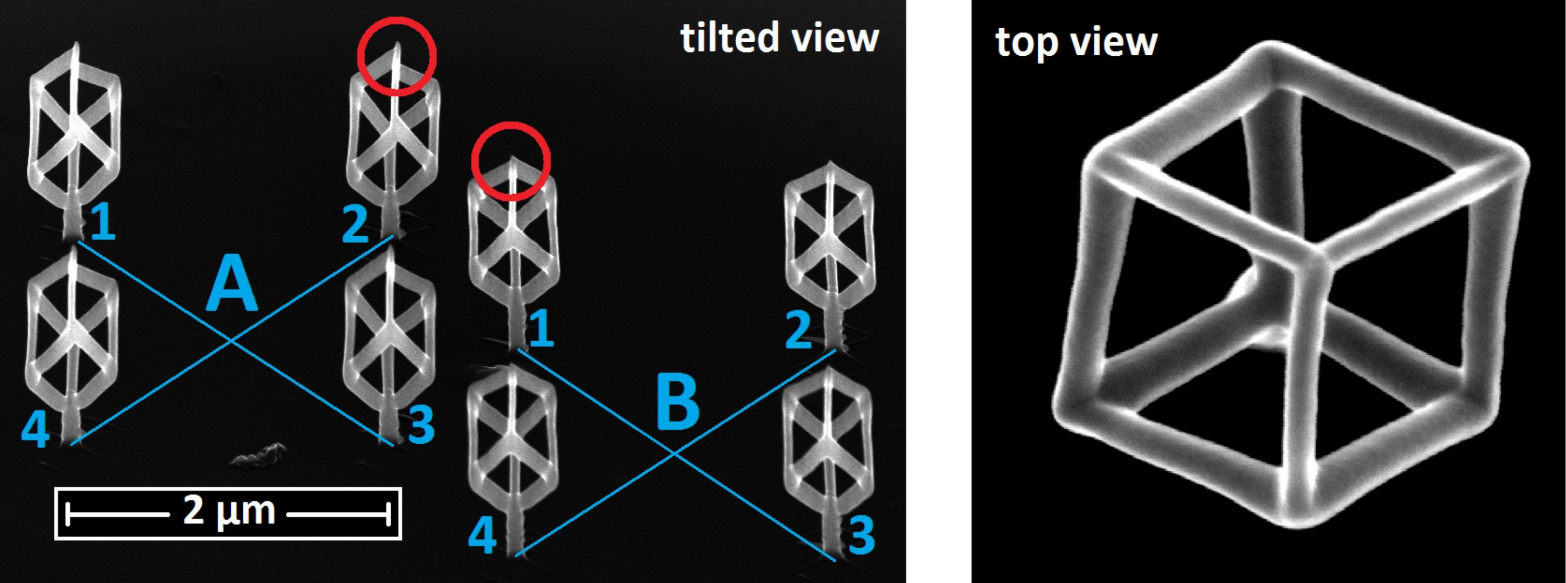
Avoiding of proximity effects: A and B are 2 × 2 arrays of cubes resting on a short pillar. They are deposited next to each other with the exact same parameters and algorithm, except of the order in which the deposition events were written. For A in every frame all three deposition events of one cube are written first before all three deposition events of the next cube follow (A1, A1, A1, A2, A2, A2, …). For B each consecutive deposition event takes place at a different cube (B1, B2, B3, B4, B1, B2, …). Note that in A the growth rate of the edges varies strongly, which is not the case in B. No shadow-avoiding and no height-correction algorithm were used for A and B. From the top view of a similar cube it is apparent that a planar intersection of one cube contains three or six points. Precursor: HCo_3_Fe(CO)_12_, high-resolution mode, beam current: 13 pA.

At first we show the improvement of using the (fast) angle-sorted proximity avoiding algorithm (asPAA) instead of no proximity algorithm in Figure 8. One can see the influence of order of DEs within a frame. The writing order has a distinct effect on the actual growth rate of an individual edge, since proximity effects and insufficient precursor replenishment times both lead to a reduction of available precursor. Figure 8 shows two 2 × 2 arrays of cubes, denoted as A and B, which were both written with the same entries in the pattern file but with a different order of these entries. For A no proximity correction was used in the pattern file generation, whereas an asPAA was used for B. As each cube has its threefold space diagonal perpendicular to the substrate surface, each frame consists of three or six DEs per cube. For A, all three DEs of a cube within one frame were written in sequence before the next cube was addressed. We indicate the corresponding writing order as A1, A1, A1, A2, A2, A2, …, where the number refers to the cubes 1, 2, 3, and 4. In contradistinction, for B the writing order was B1, B2, B3, B4, B1, B2, …. As is apparent from the figure, for A the edge growth rate shows pronounced variations, which is not the case for B (see red circle marks in Figure 8).

**Figure 9:**
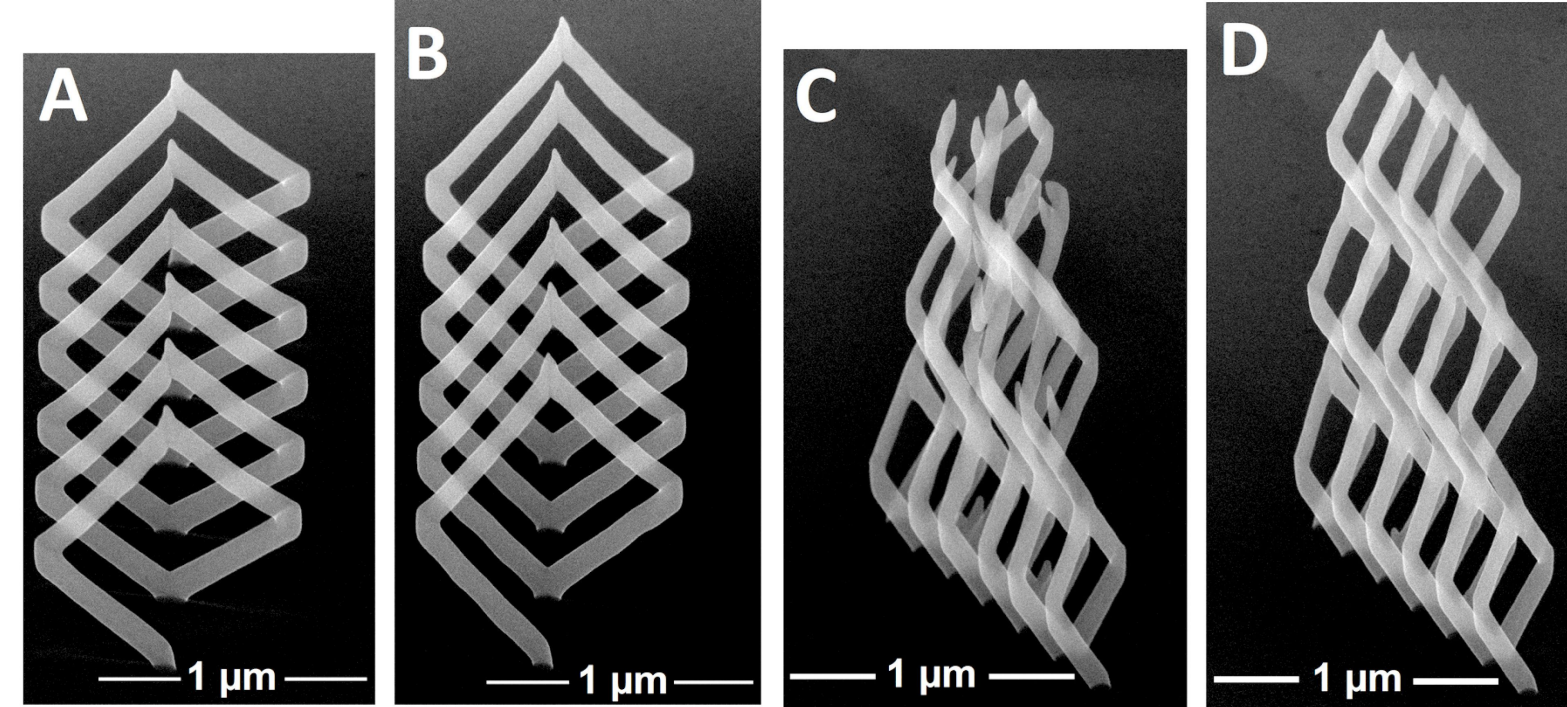
Comparison of the angle-sorted proximity avoiding algorithm (asPAA) and the best permutation PAA (bpPAA). All images are taken at 52° tilt angle. The pattern files for A and C were generated with the asPAA, for B and D with the bpPAA. Note the different height mismatches of the left and right edges of A, in contrast to B. C and D are three horizontally aligned coils stacked on top of each other. B (1.37 μm) is taller than A (1.09 μm) and D (2.46 μm) is taller than C (2.38 μm) due to more efficient use of precursor. Precursor: Me_3_CpMePt(IV), high resolution mode.

Next we present two examples which demonstrate the different performances of the (fast) angle-sorted proximity avoiding algorithm (asPAA) and the computationally more expensive best permutation PAA (bpPAA) in Figure 9. Deposits A and B in Figure 9 have been deposited with the same parameters except for the used PAA. The same is true for C and D. The pattern files for A and C were generated with the asPAA, the pattern files for B and D with the bpPAA. Besides the fact that both A and C do not comply as nicely to the target geometry as B and D, B and D are also taller due to more efficient use of precursor. All four structures were deposited in high-resolution mode on SiO_2_ employing the platinum precursor.

### 3.3 Height correction

**Figure 10:**
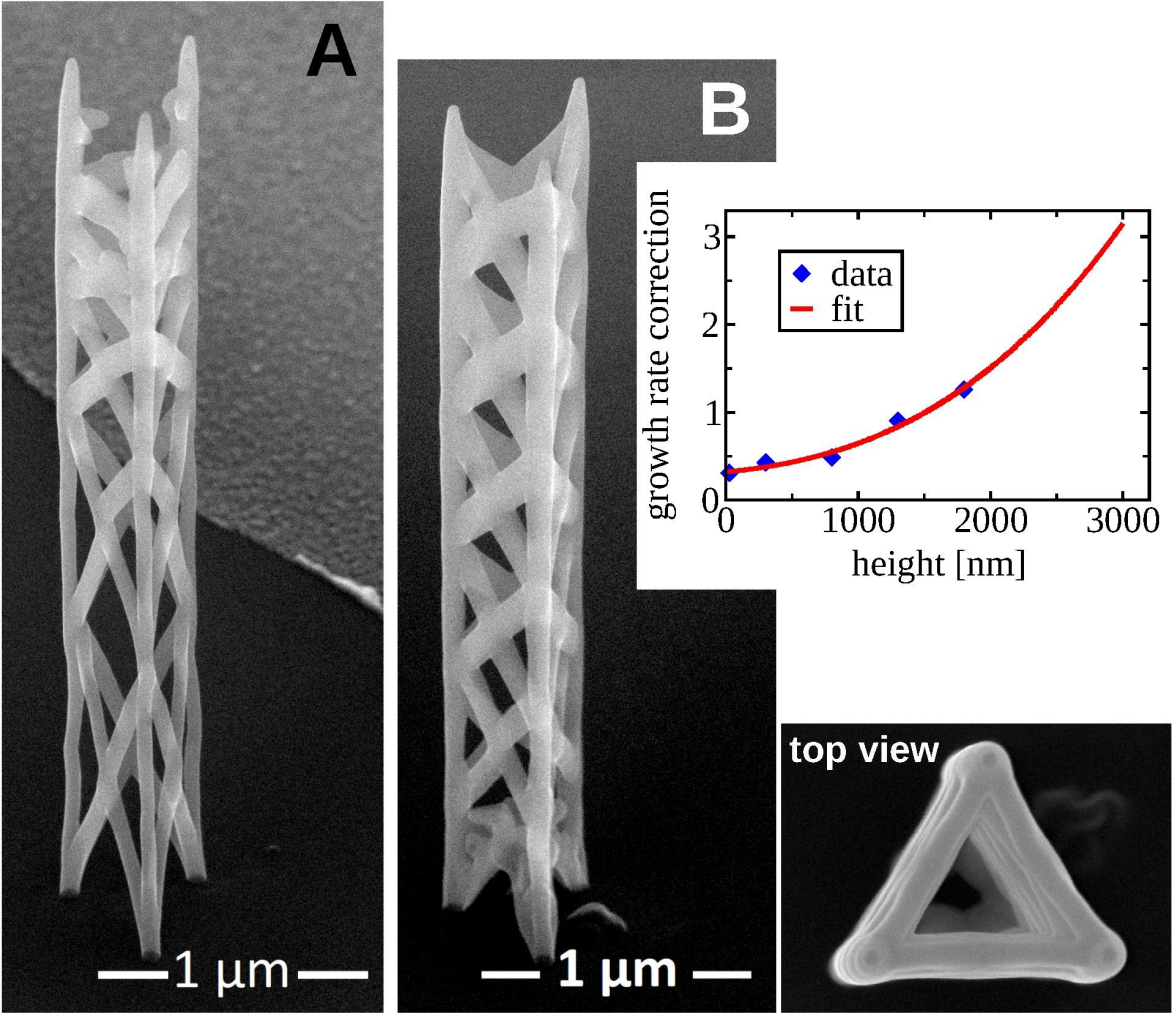
Height correction: Both left images are taken from a 52° tilted view. (A) is deposited without height correction. From height measurements of this deposit the height correction function was gained (red continuous line in the plot). With the corresponding fit coefficients the pattern file for deposit (B) was generated. Note the almost equidistant floors of (B). Especially in the upper floor levels a pronounced improvement is visible in comparison to (A). Precursor: Me_3_CpMePt(IV), high-resolution mode.

In Figure 10 we demonstrate the positive influence of the height correction. After deposit A was written without height correction, the heights of some corner vertices were measured from a SEM image and compared to the heights defined in the geometry file geof (compare with section 2.3.1). The calculated height corrections are shown as blue diamonds in the upper right plot. The corresponding fit to a polynomial function of order three is also shown in red. The coefficients from the fit have then been used in the settings file setf for deposit B. In consequence, the growth speed *s*_F_ of each edge is divided by the fit function according to the edge’s height. The resulting deposit turned out to have almost equidistant floor heights, as defined in the geof, and even the uppermost floor is quite regular, in contrast to deposit A for which the last floor is not deposited correctly at all.

### 3.4 Deposits with increased complexity

**Figure 11:**
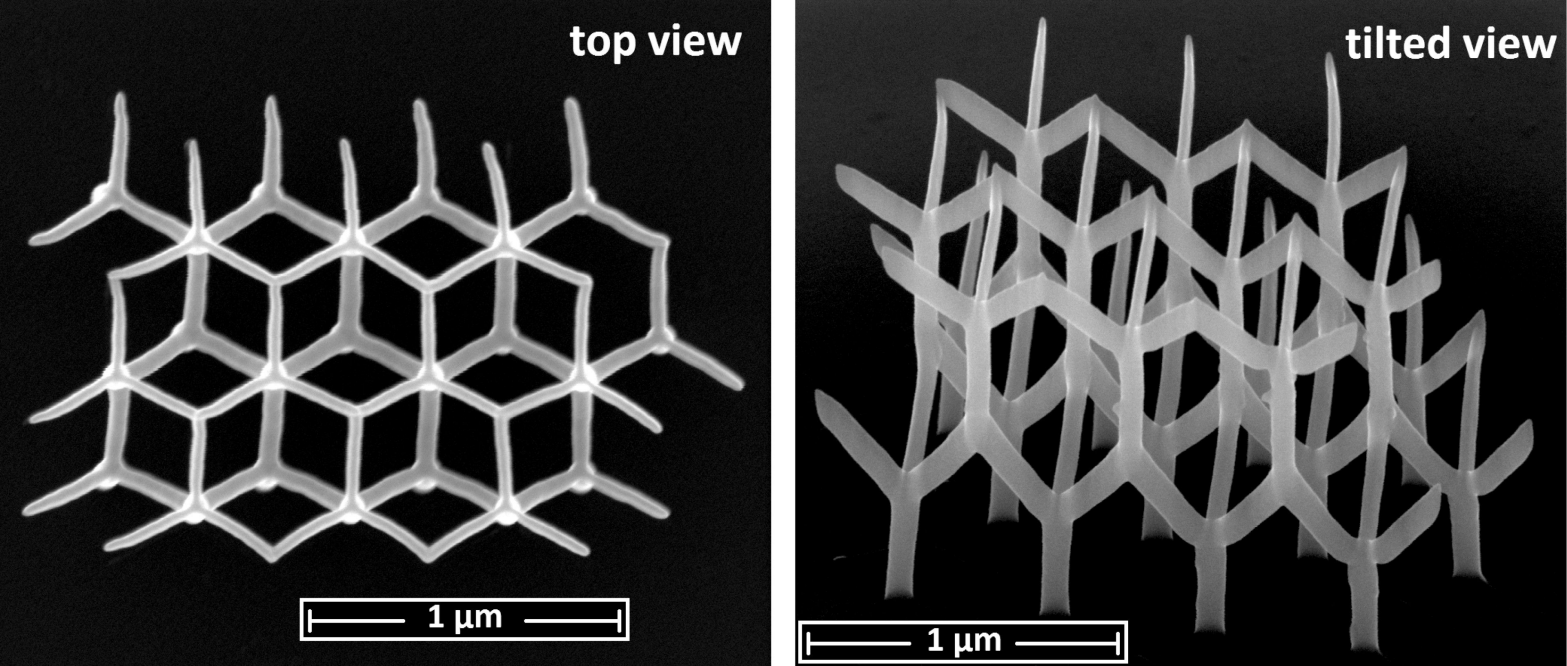
Array of trees, each consisting of a root and three branches. The roots of the trees of the second level rest on two or three connected branches of the first level. The total deposition time was 12:58 minutes. Precursor: HCo_3_Fe(CO)_12_, high-resolution mode, beam current: 13 pA. Figure first published in [25].

**Figure 12:**
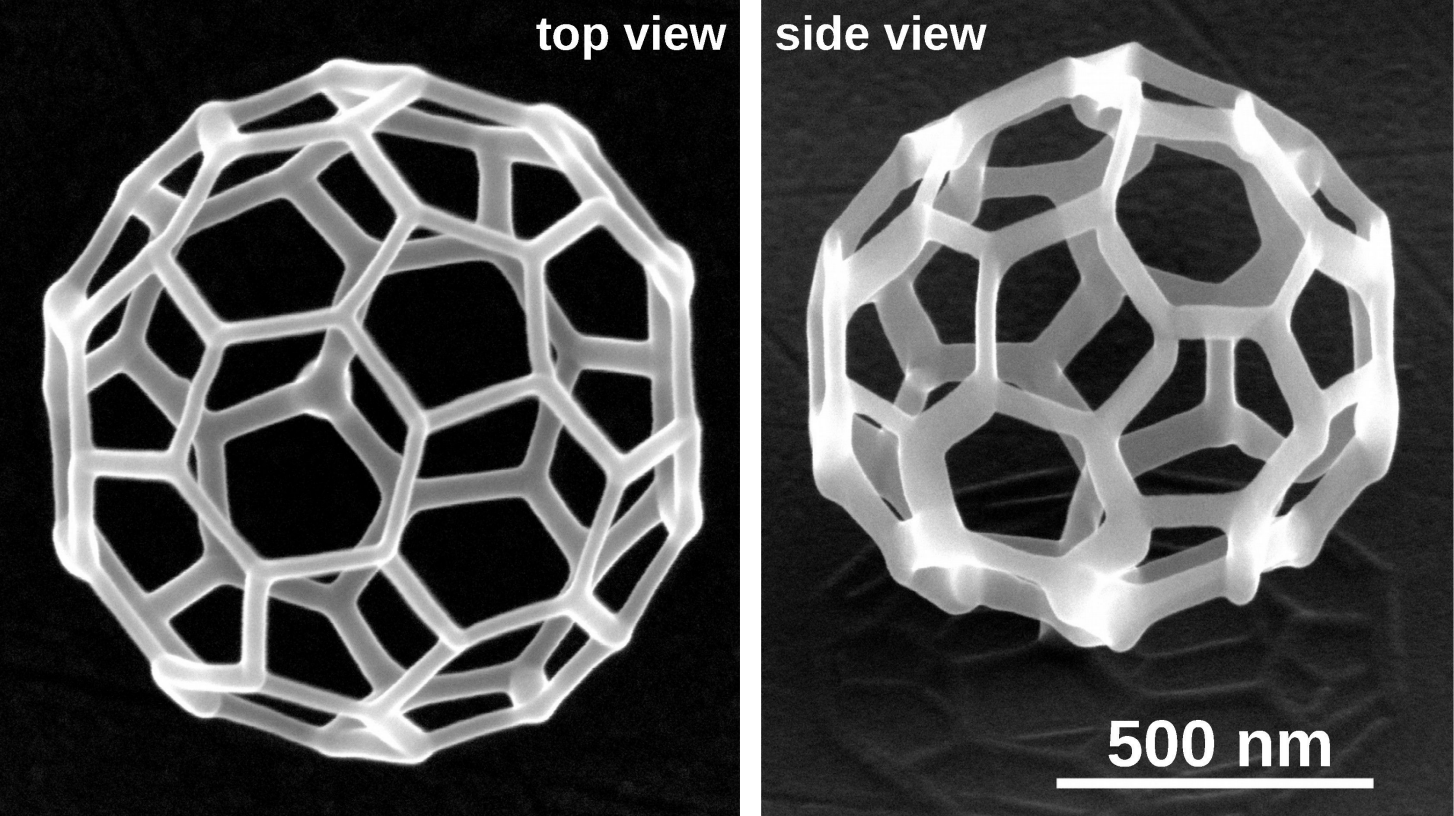
Buckyball structure in top and side view (52° tilted). Precursor: Me_3_CpMePt(IV), normal mode.

As a demonstration of the capability of the pattern file generator for more complex 3D growth, we show in Figure 11 a Co_3_Fe edge array according to a diamond lattice structure with relevance for studying frustrated magnetic interactions in 3D lattices [25]. One may also consider this structure as an array of tetrahedrally coordinated nanotrees. For this particular structure we found that the height-dependent deposition speed correction could be disabled, because for complex structures with many DEs per frame the precursor replenishment time can be already long enough if a proximity correction, i.e., an optimized writing order within a frame, is used. As a second complex example we show in Figure 12 the buckyball motif (like in the C60 fullerene molecule). Since there is a very large range of different edge inclination angles in this structure it is especially challenging to get nicely connected edges.

#### 3.4.1 Two-material heterostructures

**Figure 13:**
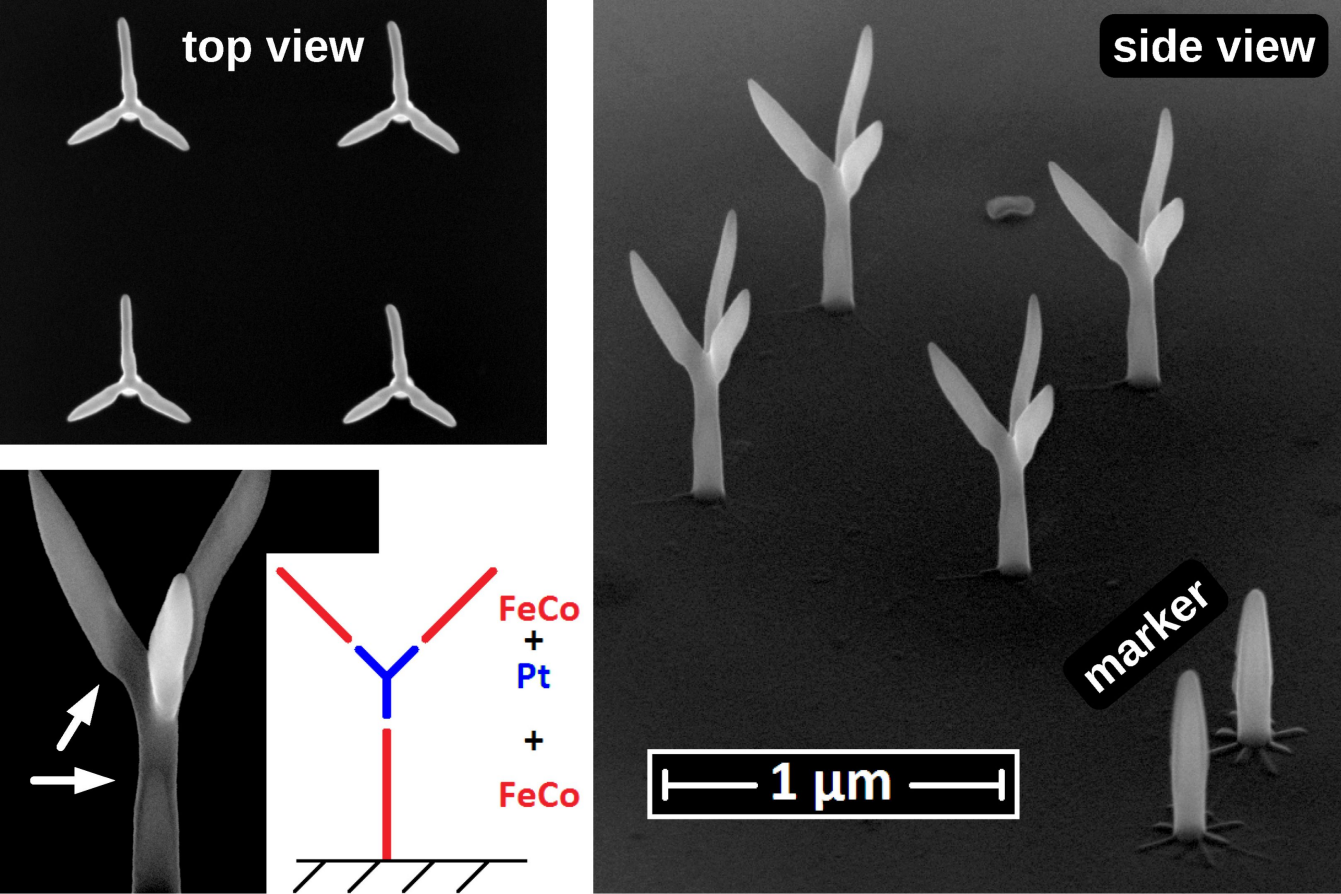
2 × 2 array of nanotrees consisting of a root and branches deposited with the CoFe precursor and a connecting node in the middle of each tree deposited with the Pt precursor. In the bottom right corner two pillars, serving as markers, are visible. On each pillar base a thinly-defined star pattern is visible, which was deposited and used for aligning the second (Pt(C)) and third (CoFe) structure elements. The pillars are part of the first pattern file. Every star is part of either the second or the third pattern file. They can be defined by a single line in the geof. Beam current: 13 pA.

In section 2.3.3 the possibility to deposit 3D heterostructures using different precursors in sequence has been alluded to. In Figure 13 we present an example of a 2 × 2 array of nanotrees, where the edges consist of Co_3_Fe but the central vertex segment is made of nanogranular Pt(C). In this case, replacing the vertex segment by a non-ferromagnetic material is beneficial for reducing the complexity of the magnetization distribution in diamond-like 3D lattice structures [25].

### 3.5 Performance

Table 1 gives an overview of the execution times of the pattern file generator code for selected examples of this section. The program was executed in a single thread on a laptop with Intel i7-2630QM processor and 8 GB 1333 MHz DDR3 RAM.

**Table 1:** Execution time of the pattern generator for selected examples of generated pattern files. All pattern files were generated using parameter settings for the Pt precursor. #DE specifies the total number of deposition events of the pattern file, #*f* the total number of frames and PAA the angle sorted (as) or best permutation (bp) proximity avoiding algorithm. Note that the single coils from Figure 9A and B are two times larger in *x*-, 2.5 times larger in *y*- and roughly two times larger in *z*-direction than each coil of the 3-layered coils from Figure 9C and D. Although Figure 9B has less DEs and frames than Figure 9D, the computation time for B was longer due to an approximately 18% higher number of DEs per frame in B than D (the higher coils in D have less turns). More DEs per frame make the bpPAA slower, since the number of possible permutations is larger. For the generation of the buckyball a smaller *n*_survivors_ was used than for the coils which results in a shorter execution time.

Object	Figure	Gen. time [s]	#DE	#*f*	PAA	Dep. time [s]

coil	Figure 9A	1.2	153 223	13 255	as	153
coil	Figure 9B	258.1	153 223	13 255	bp	153
3 coils	Figure 9C	1.5	207 623	21 231	as	208
3 coils	Figure 9D	244.2	207 623	21 231	bp	208
tower	Figure 10A	3.7	418 257	46 996	as	418
tower	Figure 10B	3.8	445 185	49 634	as	537
buckyball	Figure 12	108.1	120 383	16 886	bp	119

In general, the execution times of the algorithm is in the range of a few seconds up to several minutes, depending on the size of the object, the proximity avoiding algorithm (PAA) used and, especially for the best permutation PAA (bpPAA), the number of DEs within one frame. The execution time also depends significantly on the used settings (*n*_survivors_, loop number).

## 4 Discussion

The implementation of the algorithms relating to proximity corrections and height-dependent precursor coverage in section 2.3 already allow for the generation of suitable pattern files even for rather complex 3D target geometries with FEBID. Nevertheless, further improvements are possible, e.g., with regard to considering changing precursor replenishment times, to improving the edge inclination angle accuracy and to avoiding possible shadowing effects, which we will discuss in the following. We start with providing some more details concerning the physical context of the multifacetted parameter *s*_F_ introduced in section 2.1.

### 4.1 Physical context of the deposition speed *s*_F_

*s*_F_ is an interpolation of *x*_F_ and *z*_F_ dependent on the edge’s inclination angle. *x*_F_ and *z*_F_, and accordingly *s*_F_ merge several substrate-, precursor- and beam-parameter-dependent properties into one parameter. *s*_F_ reflects the length increase per deposition event DE referring to one edge. Its initial value, as specified in setf by *x*_F_ and *z*_F_, is based on the assumption of saturated precursor coverage, as is expected for deposits starting at zero height. Since the growth rate furthermore depends on the precursor flux, beam energy, the beam current, the (energy averaged) dissociation cross section and the volume of the nonvolatile part of the dissociated precursor molecule [8], *s*_F_ is also governed by these process- and precursor-specific parameters. As the effective precursor supply by surface diffusion drops with increasing height of the growing deposit, *s*_F_ needs to be adapted accordingly and this has been discussed in section 2.3.1. Here we note that the experimentally determined initial values for *x*_F_ and *z*_F_ at fixed beam energy and current can easily be an order of magnitude different for different precursors, even if those are supplied with comparable precursor flux and GIS geometry. This is mainly due to the differences in the respective dissociation cross sections, diffusion constants, average residence times and volume of the nonvolatile fractions of the precursor molecules. In our case, the initial *x*_F_ and *z*_F_ values differed by a factor of four comparing Me_3_CpMePt(IV) and Co_3_Fe(CO)_12_ as precursors for quite similar GIS geometries and precursor flux values.

### 4.2 Shadowing of directed precursor flux component

**Figure 14:**
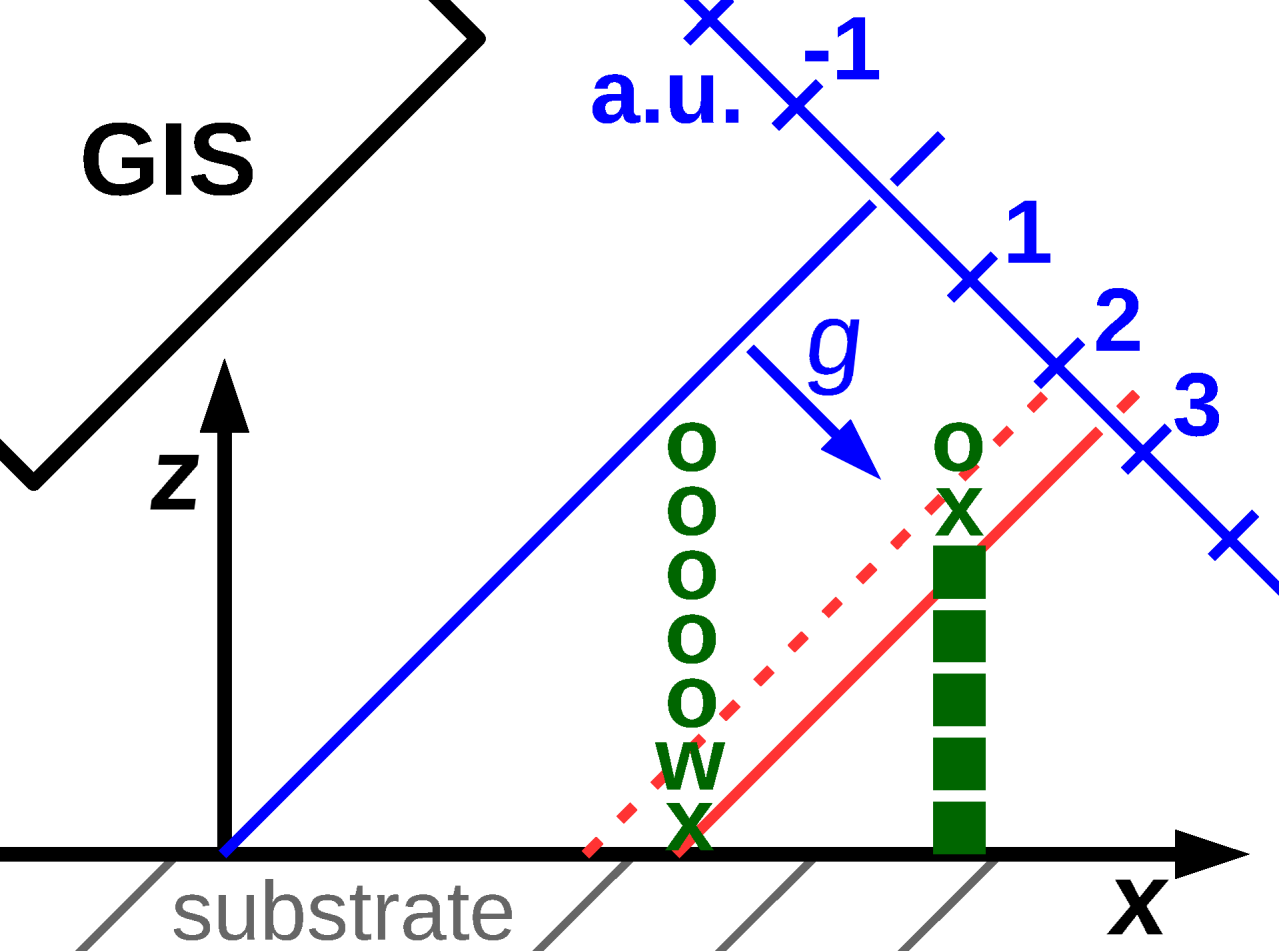
Working principle of the simple shadowing algorithm. A plane is defined parallel to the opening of the GIS needle (vector *g*) and intersecting the coordinate system’s origin (blue line). In every preceding frame the maximum distance of all processed DEs is stored (red solid line). Only the deposition events farthest from the GIS needle within one frame within a small distance range, like 5 nm (dashed red line) are processed in a given frame. Filled squares represent already deposited deposition events (DEs), ”x” are the DEs to be processed in the shown frame, ”o” are DEs too close to the GIS needle and ”w” are DEs not too close to the GIS but not reached by the corresponding edge in this frame.

In FEBID with a capillary-based GIS the precursor flux has a directional and a nondirectional component. The directional flux to the location of a DE may be impeded by the already fabricated part of the 3D target structure [31], as is schematically indicated in Figure 14. A solution to this problem must address the task of how to sort the order of DEs not just within a frame but between several frames such that shadowing effects are avoided. Here we present a simple approach that does not need any preprocessing of the geometry of the target structure defined in geof. The GIS capillary orientation determines the direction of the directional precursor gas flux component which we indicate by the normalized vector *g* = (*g_x_*, *g_y_*, *g_z_*) in the 3D coordinate system. We consider a plane oriented perpendicular to *g*. For convenience, we let this plane pass through the point (0, 0, 0) of the 3D coordinate system. For the first frame consisting of the set of DEs that belong to all active edges which have any of the initial vertices as starting vertex we determine the signed distances {δ*_i_*} of the locations {*P*_3D_} of the DEs to this plane by a simple scalar product and save the largest value δ_max_


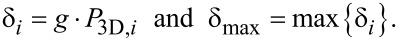


Now, for any of the subsequent frames we proceed as follows


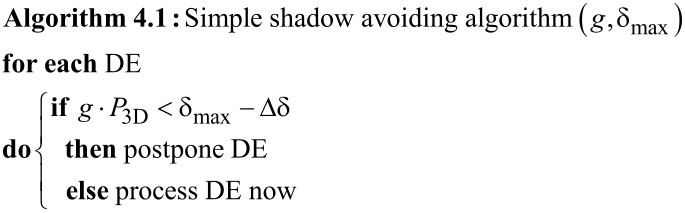


The small positive offset Δδ is set to 5 nm and its function will become apparent shortly. The set Ω = {DE} of all postponed DEs remains to be processed in the next frame. In every frame the value of δ_max_ is changed to max{δ*_i_*} with {δ*_i_*} calculated from all (processed and not processed) DEs of the current frame. By this, if none of the DEs in the given frame turn out to be processed, in the next frame at least the one DE in maximum distance to the GIS will be processed. In essence, the algorithm in conjunction with the small offset Δδ ensures that the front of DEs progresses from the locations furthest from the GIS opening towards the GIS. By this strategy, direct shadowing is effectively debarred. However, depending on the geometry of the target structure, the resulting sequence of processed DEs may not be optimal, in particular as many DEs can become shifted towards the end of the pattern file even though they would in fact not have caused shadow effects. This can be avoided with an algorithm which calculates the actual shadowing, and which will be discussed in the next section. We conclude this paragraph by noting that we did not find the need for using a direct shadow avoiding algorithm for our depositions so far. This might be due to the fact that the direct precursor flux from the GIS to the place of deposition is much smaller than the precursor supply by diffusion from the substrate.

We note that the simple shadow avoiding algorithm with a virtual vertical alignment of the GIS can guarantee that no higher DEs are written before lower ones (e.g., if one has to deal with initial vertices on both, the SiO_2_ substrate and gold electrodes on a different height).

### 4.3 Advanced shadow avoiding algorithm

The advanced approach to avoid shadowing effects is based on considering the shadow effect caused by the small amount of deposit generated by any given DE on all subsequent DEs. In order to analyze this, the pattern file generation is only done after a preprocessing step based on the geometry file. During preprocessing an array is created that contains the positions {*P*_3D_} of the DEs employing the height-dependent precursor coverage correction but with shadow and proximity avoiding disabled. In the pattern file generation, the array content is used to judge whether a scheduled DE’ will cast a shadow on the DEs waiting for later completion. This phrasing is shorthand for calculating whether an assumed cylindrical light tube of radius *r*_shadow_, aligned with its symmetry axis along the GIS axis and passing through DE’, contains any of the positions {*P*_3D_} of the DEs still waiting in line. If this is the case, DE’ is shifted to the next frame. This leads to an optimized order in the sequence of DEs in the generated pattern file, as compared to the simple algorithm, but at the cost of a significantly enhanced computation time. This is not so much caused by the necessary preprocessing but by the ”point-in-cylinder” computation for each pending DE. Our non-optimized implementation of the advanced shadow avoiding algorithm does not take advantage of the fact that the ”point-in-cylinder” calculation could be parallelized. As a consequence, the execution times are quite significant.

### 4.4 Possible further improvements

#### 4.4.1 Depth of focus

The limited depth of focus of the SEM can become an issue in high-resolution mode (large numerical aperture) with growing 3D deposit height. This is in principle easy to correct but requires that the SEM’s focus can be modified automatically as the pattern file is processed. Winkler et al. [28] suggest to use normal mode in most 3D writing scenarios.

#### 4.4.2 Refresh time

A changing growth rate as a consequence of strongly differing writing times for different frames can become an issue because of the associated difference in the precursor coverage. For target geometries with pronounced changes of the number of edges from one height level to the next, one might therefore need a corresponding refresh time correction. In our implementation this could be accomplished by adding a refresh time between one frame *f_i_* and the next *f_i_*_+1_ that becomes larger with a smaller number of DEs in *f_i_*. A more elegant solution would need to adopt *s*_F_ dynamically depending on the duration of the last frame. A fully satisfying solution may only be achieved by a free definable height correction function which is gained by a FEBID simulation.

#### 4.4.3 Edge shape

For some applications of 3D FEBID structures, the shape of the edges’ cross section can be important. From Figure 11 it is apparent that the cross sections are not circular which adds complexity to the magnetization distribution in the Co_3_Fe array. The main reason for noncircular edge cross sections is the non-homogeneous generation of secondary electrons inside the already deposited material. Any non-homogeneous secondary electron distribution within the escape depth of the secondary electrons will lead to corresponding inhomogeneous growth rates.

**Figure 15:**
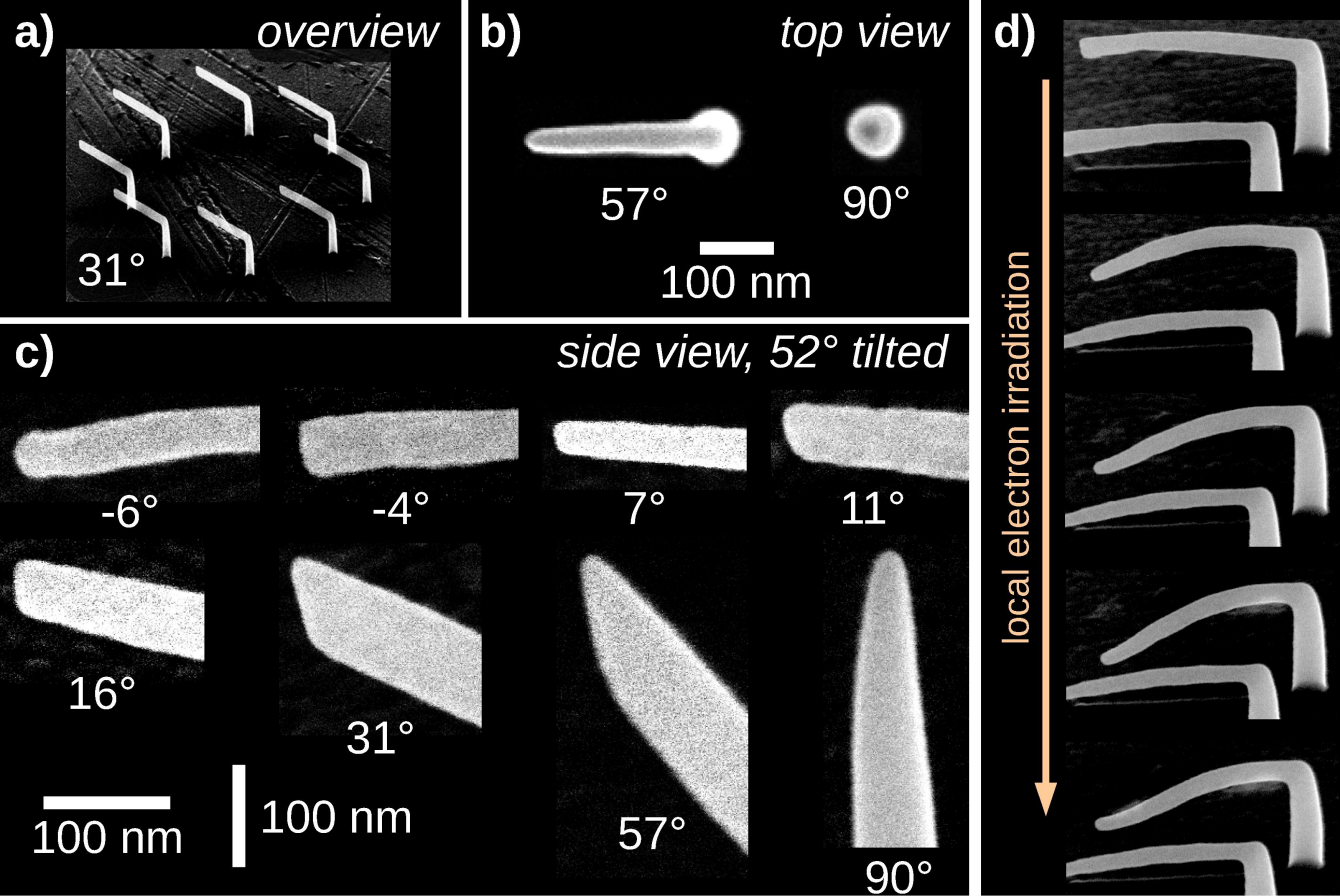
In order to investigate the shape of edge tips with different edge inclination, deposits like shown in a) were prepared. They consist of eight identical elements, written in parallel, in order to guarantee sufficient refresh time for precursor replenishment. b) Tip shapes as viewed from top. Since the deposits’ shape can be modified by the image acquisition, no images for edges with smaller inclination angles are shown. c) Tip shapes from a 52° tilted view. The exact shape of a tip will influence the ratio of secondary electrons leaving the edge’s top and side surfaces. An improved inclination-angle-dependent interpolation function for the deposition speed *s*_F_ could use this information. d) Influence of electron irradiation on the shape of 3D structures. Even the relatively small electron dose applied during image acquisition of a deposit can lead to shape changes in quite dramatic ways. Precursor: Me_3_CpMePt(IV), normal mode.

In order to improve the interpolation function of *s*_F_ between *x*_F_ and *z*_F_ to get a better inclination angle definition (see section 3.1), one should take the shape of the tip of a deposited edge into consideration, which also determines the ratio of secondary electrons leaving the edges top and side surface. A selection of possible tip shapes is shown in Figure 15c.

### 4.5 Comparison to other 3D pattern generation software approaches

To the best of our knowledge, only one other software approach for 3D FEBID pattern generation has been published, namely by Fowlkes et al. [27] in collaboration with Winkler et al. [28]. Here we briefly outline the similarities and differences between our approach and that published in [27].

#### 4.5.1 Height-dependent growth rate correction

Our approach compensates for height-dependent growth rate changes by introducing a height-dependent deposition speed correction function and does not require modifications in the geometry definition file. In [27] the geometry of the target structure is modified by the user to compensate for height-dependent growth rate changes.

#### 4.5.2 Proximity effect prevention

To our understanding, in [27] no automatic order optimization of the deposition events has been implemented. Here, we describe such automatic routines (angle-sorted and best-permutation) which are both shown to be effective in reducing proximity effects.

#### 4.5.3 Inclination angle of edges

In [27] arrays of simple reference structures created with different deposition speeds and correspondingly different inclination angles are used to measure the resulting inclination angles. This is then used as calibration data. Here, we calibrate only for horizontal and vertical edges and interpolate for other inclination angles using an interpolation function.

#### 4.5.4 Constant pitch versus constant dwell time

The inclination angle of any edge is determined by the deposition speed, which is the ratio of the point pitch and dwell time. There are three possibilities to change the deposition speed: Changing the dwell time at constant pitch, as done in [27], changing the pitch at constant dwell time (our approach), or changing both. Note that the choice of one of these strategies is independent of the previously discussed different possibilities of how to achieve well-defined inclination angles (previous subsection).

Neither of the two approaches leads to a better spatial resolution at low magnification, since both the value for the fixed dwell time as well as for the fixed pitch can be adjusted accordingly. For both approaches the digital to analog converter (DAC) of the pattern generator of the SEM will work with the smallest incremental steps available at low magnifications.

Although the inclination angle of an edge depends on the averaged deposition speed with a high accuracy, typically smaller than 0.02 nm (Figure 6), the DAC does not need to achieve a pitch of this precision in every frame, but only in average over several frames (we estimate our achievable resolution to be 0.3 nm at a HFW of 4590 nm). As a consequence, although an edge proceeds to grow in a given direction, the DAC will address the exact same position in two following frames (this will be always the case for vertical pillars). One can interpret this as a ”doubled dwell time”, but with the advantage that there is a refresh time between the first dwell time and the second. This ”multiple dwell time” will also not be in effect for all DEs of the affected edge to the same extent, but only transitional for some DEs, depending on the edge’s inclination and the used HFW.

Our fixed dwell time approach makes sure that all deposition events will experience very similar precursor replenishment and dwell times, thus keeping the growth regime stable (!) over the full deposition, most specifically independent of the inclination angles of the edges in each frame.

The dwell times necessary in [27–28] (50 ms, corresponding to approximately 70° inclination) seem to be quite long compared to typical dwell times in FEBID for 2D structures, especially considering limited precursor replenishment dynamics. Finally, as a side remark, a fixed pitch as used in [27] requires an infinite large dwell time for vertical edges (pillars).

#### 4.5.5 Software design

Whereas the implementation described in [27] requires a suitable Matlab environment, our implementation in native C++ is notably independent from third party software. A graphical user interface is not provided within our implementation, whereas this is an essential ingredient in [27], in particular with respect to the edge-inclination problem as discussed above. As another minor difference we mention that in [27] the user is asked to define ”exposure levels” in order to tell the program which edges to write in parallel. In our solution, this task is performed parenthetically by our algorithm.

## 5 Conclusion

In summary, we have presented a pattern file generation program that implements different correction algorithms for the generation of optimized pattern files for the reliable fabrication of 3D nanoarchitectures of various complexity levels by focused electron beam induced deposition. The implementation solves the main issues one encounters in 3D nanofabrication by FEBID, namely proximity effects and height-dependent precursor coverage. Additionally, shadowing of the directed precursor gas flux component can be considered. We demonstrated an alternative approach for depositing edges with a defined inclination angle in comparison to [27–28]. We also presented selected examples of 3D structures of different complexity levels to illustrate the effectiveness of our implementation. The use of only standard C++ libraries resulted in code independent of third party software and in fast execution times. We hope to contribute to the future development of 3D FEBID pattern generation software and to provide a useful software tool to others that venture to start into the fabrication of 3D nanoarchitectures for various application fields. The compiled pattern file generator is available on request (please contact corresponding author) for noncommercial usage.

## Supporting Information

Explains the input and output files of the pattern generator program in detail (see also Figure 1). The parameters of the settings file setf are defined. The structure of the geometry file geof and the generated pattern file are shown. The usage of the illustration file and the description file is demonstrated.

File 1 Supporting information.
