# Supplementary material for: Pattern generation for direct-write three-dimensional nanoscale structures via focused electron beam induced deposition
Source: Beilstein J Nanotechnol. 2018 Sep 27;9:2581–98. doi: 10.3762/bjnano.9.240 (PMC6176821; doi:10.3762/bjnano.9.240)
Supplement: File 1 — Supporting information. [file Beilstein_J_Nanotechnol-09-2581-s001.pdf]

Supporting Information

for

Pattern generation for direct-write three-dimensional nanoscale structures via focused electron beam induced deposition

Lukas Keller[1] and Michael Huth*[1]

Address: [1]Institute of Physics, Goethe University, Max-von-Laue-Str. 1, 60438 Frankfurt am Main, Germany

Email: Michael Huth - michael.huth@physik.uni-frankfurt.de

* Corresponding author

1 Files

Necessary input for the *pattern file* generator are the settings file *setf* and the geometry file *geof*. By separating the target geometry from all other process-specific parameters, a large degree of flexibility is gained. Also, no modifications of the program environment are necessary if a graphical user interface is implemented to provide a convenient possibility to enter process parameters to be written to *setf* or if a computer-aided design (CAD) tool is used to simplify the design of the target geometry. In this case a simple filter program can generate the necessary geometry file *geof* from the CAD-specific file.

1.1 Settings file

The file *setf* specifies the following parameters

HFW – Horizontal field width in units of nm

z_F – in units of nm per frame at the height position of the substrate surface. z_F depends on the used precursor, GIS settings, beam parameters, dwell time, refresh time and the geometry defined in *geof*. The value of z_F is gained from calibration experiments as described in section 2.2 and 3.1, main text.

x_F – in units of nm per frame. Its value is gained from a deposition like shown in Fig. 6, main text.

Initial t_d – in units of 100 ns with a typical value of 10 000 (which corresponds to 1 ms)

$\{\alpha_n\}$ – coefficients for the polynomial fit (3rd order) needed for the height correction, see section 2.3.1, main text. $\{\alpha_n\} = \{1, 0, 0, 0\}$ effectively disables the height correction.

Maximum edge length l_{max} – for the initial subdivision of edges, in units of nm. A typical value is 20

Shadow avoiding algorithm to use – 0: none, 1: simple (see section 4.2, main text), 2: advanced (see section 4.3, main text)

GIS angles – polar angle P measured against the xy-plane, i. e. $P = +90°$ is perpendicular to the substrate, and azimuthal angle A around the z-axis with $A = 0°$ points in negative y-direction. For $A < 0$ the GIS comes from the left side. P and A in degree.

Shadow radius r_{shadow} – radius parameter in nm needed for the advanced shadowing algorithm, see section 4.3, main text. A typical value is 80

Proximity avoiding algorithm to use – 1: angle sorted proximity avoiding algorithm (asPAA), 2: best permutation PAA (bpPAA) with initial guess as *DE*s are processed internally by the program (not recommended), 3: bpPAA with result of asPAA as initial guess

asPAA: angle basis – a value of 3 means a triangular like pattern should be sought, a value of 4 causes a quadrangle like pattern, and so on

bpPAA: nr of survivors – maximum number of orders which every generation of orders should have

bpPAA: nr of loops – number of generations until final order is chosen

bpPAA: sigma – distance between two *DE*s at which the cost drops to a $1/e^{th}$ of the zero-distance cost

bpPAA: prevent duplicates – 1: an order is not allowed to occur more than once in a generation (recommended)

1.2 Geometry file

The geometry file *geof* defines the 3D target structure by a list of 3D vertices and associated edges. The vertex coordinates are understood to be given in nm units. The vertices are automatically numbered. Those vertices which represent the *initial* vertices, from which the target 3D structure is eventually generated as the *pattern file* is processed, are listed separately by their respective indices. The edge list states for each edge the indices for the respective start and end vertex. The coordinates

of each vertex have to be separated by any number of spaces or tabs, as well as the indices of each edge definition. A simple example for a geometry file appears in Fig. S1.

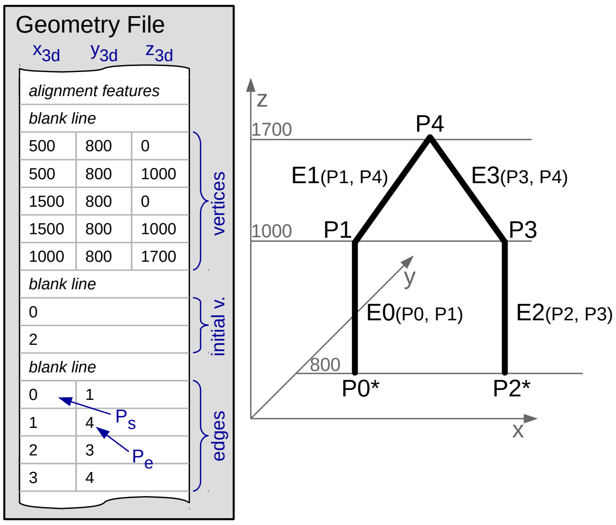

Figure S1: Structure of the geometry file *geof*. The *geof* begins with special commands to request alignment features (see section 2.3.3, main text), which can be omitted. Then in any case there has to be one blank line. Next the coordinates of all vertices are given in nm, followed by a blank line. Next the initial vertices are defined, from which the whole 3D structure will be constructed from, followed by a blank line. Finally the start and end points of each edge are specified. On the right side of the graphic the corresponding 3D structure is shown.

In the current version of the program there are the following commands available for using alignment features. Each command has to be in an extra line. Do not add additional blank lines between these commands. All spacial parameters are given in the unit nm. Parameters have to be separated by any number of spaces or tabs. Also the "refresh" command, which is not a structure for alignment purposes, has to be given at the beginning of the *geof*.

cornerPoints upperLeft.x upperLeft.y lowerRight.x lowerRight.y – Generates four dwell points at the corners of a rectangle, spanning from the upper left to the lower right coordinates. The four dwell points use the shortest dwell time possible and are addressed at the beginning of the *pattern file*. Usage example: "cornerPoints 500 500 4000 4000"

star center.x center.y center.z radius pitch dwell duration – Generates a 2D star at the position of center, with a radius, pitch and dwell time (in units of 100 ns) as specified. The

exposure of the star will take "duration" seconds at the beginning of the *pattern file*. Usage example: "star 3000 3000 -1000 120 10 100 15"

cross center.x center.y center.z radius pitch dwell duration – Generates a 2D cross at the position of center, with a radius, pitch and dwell time (in units of 100 ns) as specified. The exposure of the cross will take "duration" seconds at the beginning of the *pattern file*. Usage example: "cross 3000 3000 -1000 120 10 100 15"

refresh pos.x pos.y size.x size.y pitch dwell afterHowMany nrOfPoints – In order to increase the refresh time between deposition events, a rectangle can be defined by it's position and size. It will be addressed by the electron beam after a number ("afterHowMany") of deposition events using a pitch and dwell time (in units of 100 ns) as defined. "nrOfPoints" specifies how many points of the rectangle should be addressed before continuing with the actual 3D structure. This refresh rectangle is special, since it is written parallel to the defined 3D structure, but does not participate in (and thereby disturb) any proximity avoiding algorithm! Usage example: "refresh 4000 3000 200 100 5 10000 137 4" will write after every 137th deposition events of the 3D structure 4 dwell points with each 1 ms dwell time at the refresh rectangle.

In the current version of the program there is one extra command which can be placed a line before any edge definition line:

set px *** – changes the x_F value specified in the *setf* for all following edges to ***. This command is used for generating *pattern files* for 'pitch-calibration-structures'. Usage example: "set px 0.20 *linebreak* 0   1 *linebreak* set px 0.25 *linebreak* 3   4 *linebreak*"

1.3 Generated pattern file

The generated *pattern file* starts with a header that provides SEM-software specific parameters, such as the resolution of the SEM pattern generator, the number of executions of the whole *pattern file*, which will be in most cases 1 execution, and the total number of *DE*s. The bulk of the ASCII encoded

pattern file consists of a list of entries of three numbers corresponding each to a *DE*, namely the x- and y-coordinates from $P_{2D} = \{x_{2D}, y_{2D}\}$ in the range $[0, 2^n)$, referring to the HFW, and the dwell time t_d in units of 100 ns.

1.4 Auxilliary files

In order to be able to validate the generated *pattern file* before performing a deposition, a *validation file* is generated that contains the coordinates of all *DE*s with the z-coordinate replaced by the frame number in which the *DE* was output. The *validation file* is formatted such that it can be directly loaded and displayed by the program *Gnuplot* using the following command

```
gnuplot> splot "streamFileAsGnuplot.txt"
```

or, to obtain a fancy colored result as shown in Fig. S2 (this was tested with gnuplot version 5.0)

```
gnuplot> set palette rgbformulae 22,13,-31
gnuplot> set xyplane relative 0.1
gnuplot> set view 75,345
gnuplot> set zlabel "Frame #"
gnuplot> splot "streamFileAsGnuplot.txt" with points palette
```

A short note to the usage of *Gnuplot*: Since the *patternfile* will usually contain quite big data sets, scaling the plot-window might be more practical after the command "plot x" was executed.
In addition, a *description file* is generated which lists all current settings from *setf*.

2 Execution of the pattern generator program

Please execute the command line program meanwhile the settings file (called "settings.txt") and the geometry file (called as defined in the settings file parameter "CoordinateInputFileName") are in the same folder as the program.

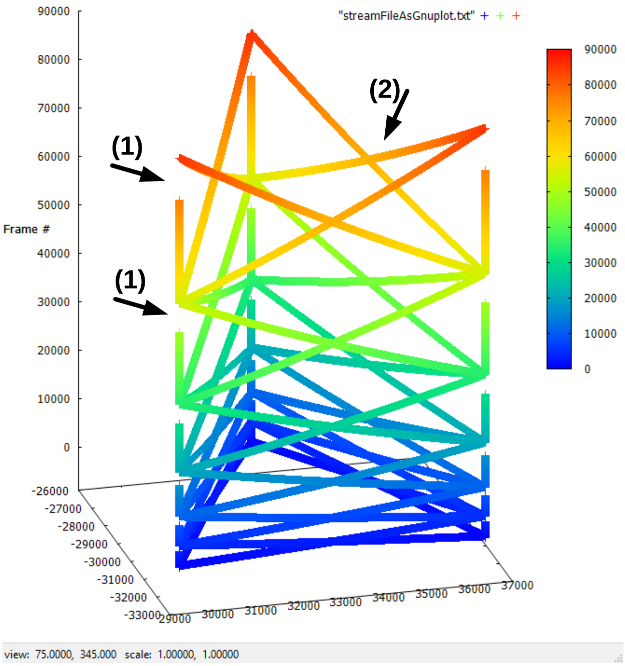

Figure S2: Output of the program *Gnuplot* for the *patternfile* which was used for depositing the height corrected tower (Fig. 10, main text). The x and y axis display the (x,y) integers given to the pattern generator of the SEM, the y values being negated. The z axis shows the frame number in which a *DE* was output. The gaps in the structure, indicated by the arrows (1), illustrate that an edge will start to be processed after it's start point is "reached". This start point is the end point of other edges. It will be "reached" only after all edges ending in this point reached it. Also the increasing height of consecutive levels of the tower can be seen clearly. This corresponds to the fact that the real growth rate reduces at higher levels and therefore more frames per level have to be spent (height correction). Most pronounced in the uppermost level, indicated by the arrow (2), the slope of the edges within one level increases. This is possible by automatically generating sub-edges of the user defined edges. Each sub-edge is linear between it's start and end point.
